# Identification of muscle weakness in older adults from normalized upper and lower limbs strength: a cross-sectional study

**DOI:** 10.1186/s13102-021-00390-1

**Published:** 2021-12-18

**Authors:** Pedro Pugliesi Abdalla, Lucimere Bohn, Leonardo Santos Lopes da Silva, André Pereira dos Santos, Marcio Fernando Tasinafo Junior, Ana Claudia Rossini Venturini, Anderson dos Santos Carvalho, David Martinez Gomez, Jorge Mota, Dalmo Roberto Lopes Machado

**Affiliations:** 1 College of Nursing at Ribeirão Preto, University of São Paulo, Ribeirão Preto, SP Brazil; 2 Faculty of Sports, University of Porto, Porto, Portugal; 3 School of Physical Education and Sport of Ribeirão Preto, University of São Paulo, Ribeirão Preto, SP Brazil; 4 Physical Education Course, Paulista University, São José do Rio Preto, SP Brazil; 5 School of Medicine, Autonomous University of Madrid, Madrid, Spain; 6 Faculty of Phycology, Education and Sport, University Lusófona of Porto, Porto, Portugal

**Keywords:** Allometrically scaled, Disability, Evaluation, Frailty, Function/functional status, Measurement, Sarcopenia, Scaling

## Abstract

**Background:**

To propose cut-off points for older adults’ weakness for upper and lower limbs muscle strength normalized by body size with the ratio standard/muscle quality and allometric scaling.

**Methods:**

Ninety-four community-dwelling older adults (69.1% women) were assessed for 49 body-size variables (anthropometry, body composition and body indexes), handgrip strength (HGS), one maximum repetition measurement for knee extensors (1RM), isokinetic knee extension peak torque at 60°/s (PT), and six-minute walk test (6MWT). Ratio standard or muscle quality (muscle strength/body size) and allometric scaling (muscle strength/body size^b^; when ^b^ is the allometric exponent) were applied for body-size variables significantly correlated with HGS, 1RM and PT. Cut-off points were computed according to sex based on mobility limitation (6MWT < 400 m) with ROC curve and Youden index.

**Results:**

Absolute HGS, 1RM and PT cut-off points were not adequate because they were associated with body size (r > 0.30). But it was corrected with muscle strength normalization according to body size-variables: HGS (n = 1); 1RM (n = 24) and PT (n = 24). The best cut-off points, with the highest area under the curve (AUC), were found after normalization for men: HGS/forearm circumference (1.33 kg/cm, AUC = 0.74), 1RM/triceps skinfold (4.22 kg/mm, AUC = 0.81), and PT/body mass*height^0.43^ (13.0 Nm/kg*m^0.43^, AUC = 0.94); and for women: HGS/forearm circumference (1.04 kg/cm, AUC = 0.70), 1RM/body mass (0.54 kg/kg, AUC = 0.76); and PT/body mass^0.72^ (3.14 Nm/kg^0.72^; AUC = 0.82).

**Conclusions:**

Upper and lower limbs muscle weakness cut-off points standardized according to body size were proposed for older adults of both sexes. Normalization removes the effect of extreme body size on muscle strength (both sexes) and improves the accuracy to identify weakness at population level (for women, but not in men), reducing the risk of false-negative/positive cases.

**Supplementary Information:**

The online version contains supplementary material available at 10.1186/s13102-021-00390-1.

## Background

Muscle weakness is a natural muscle strength loss occurring along aging, and it predicts older adults’ increased risk of hospital admissions, depression, fractures and premature mortality [1–3]. Muscle weakness can predict functional disability (i.e., difficulty to perform instrumental and basic activities of daily living-ADL) like as mobility limitation [4], which is even more important than multimorbidity to forecast mortality amongst older adults [5]. As a consequence of its predictive ability, muscle weakness was used to identify geriatric syndromes such as dynapenia [6], frailty [7] and sarcopenia [8].

Muscle weakness is normally measured using muscle strength tests such as handgrip (HGS) or leg extension strength [8]. The current values to identify muscle weakness are based on absolute (non-normalized) muscle strength results [7, 9–17] or dividing absolute results by a body-size variable (ratio standard) such as body mass [18, 19] or by some body composition component, like lean tissue (muscle quality) [20–22]. The identification of weakness based on absolute muscle strength cut-off points may be inaccurate for lighter body mass and shorter height older adults [23–25]. In fact, the absolute values characterize lighter and shorter body size older adults as having muscle weakness, even if they sustain their instrumental and basic ADL [26]. This is a false positive muscle weakness diagnostic, that frequently leads to an unnecessarily utilization of public health resources, contributing to health burden [27]. Another topic that merits consideration is the inaccuracy of the ratio standard/muscle quality procedure because it overestimates the real strength of light/short older adults and underestimates it for tall/heavy ones [26]. These limitations are a consequence of the nonlinear relationship between muscle strength and body-size variables [23–25]. To overcome these constraints, the utilization of allometric scaling, that contemplates power and sensitivity in the nonlinear relationship between muscle strength and body size with the allometric exponent (^b^) might represent an adequate option [23–26].

Previous studies reported already the power function ratio in older adults between HGS and body-size variables as body mass (^b=0.63^ or ^0.40^ or ^0.31^) [23–25], height (^b=1.84^) [24] and fat-free mass (FFM) (^b=0.46^) [24] and between leg extension strength and body mass (^b=0.67^ or ^0.69^ or ^0.72^ or ^0.74^ or ^0.96^) [26, 28]. Indeed, scaling HGS by body size (example: HGS/heigth^1.84^) removes the effect of body size on muscle strength [24], but the scaling muscle strength by body size to determine muscle weakness cut-off points has not been considered from HGS and knee extension in isokinetic dynamometer, excepting the one maximum repetition measurement for knee extensors (1RM) scaled to body mass [26]. Besides, important body-size variables related to mobility and ADL (e.g. fat mas [29], FFM [30] and leg length [31]) were not utilized to scaling muscle strength and create muscle weakness cut-off points.

Thus, our objective is to propose cut-off points for older adults’ weakness with upper and lower limbs muscle strength normalized by body-size with the ratio standard/muscle quality and allometric scaling. We hypothesize that the normalization of muscle strength by ratio standard/muscle quality and allometry can be a way to approach muscle strength regardless of body size, which should reduce the risk of bias in identifying false-positive cases of vulnerable older people.

## Method

### Design and study population

This is a cross-sectional study conducted from October 2016 to May 2017 at the University Hospital of Ribeirao Preto School of Medicine, University of São Paulo, Brazil (HC-FMRP-USP). The study was approved by the HC-FMRP-USP institutional review board (CAAE: 54345016.6.3001.5440). Older adults were voluntarily recruited and assigned an informed consent. This manuscript followed the guidelines from The Strengthening the Reporting of Observational Studies in Epidemiology (STROBE) conference list [32].

The sample consisted of 94 community-dwelling older adults (≥ 60 years old, 69.1% women) recruited in projects for older adults of USP and in health community services. Inclusion criteria were ≥ 60 years old, walk independently, absent limitation to execute all procedures, acute infections, cancer diagnosis, hip or knee prostheses, unstable cardiovascular condition, stroke sequelae, tumors, and weight loss > 3 kg in the last three months. The exclusion criteria were discontinuity in the study and cognition impairment (assessed by Mini Mental State Examination).

A sample size calculation (n = [ZySD/ε]^2^) [33] with trust level (Zy = 0.95), greater compatible population variability founded in the literature (SD of 1RM: ± 19.96 kg) [34, 35] and maximum desired error (ε ≤ 8.0 kg) was performed and identified a minimum sample size of n = 24 for each sex.

### Procedures

**Fig. 1:**
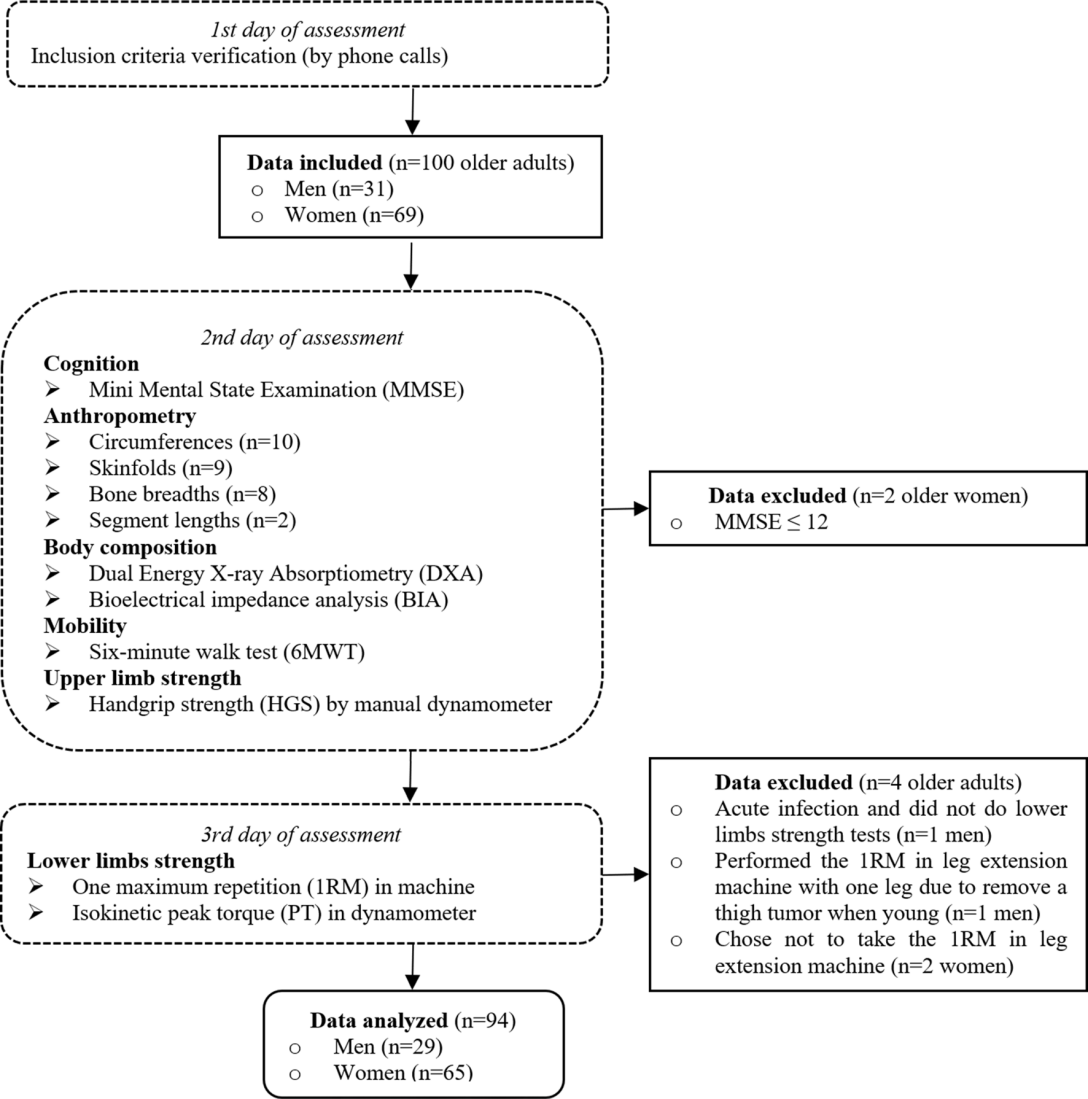
Study phases and data from older adults included, excluded, analyzed and procedure flow

A multidisciplinary health team (nurses, nutritionists, pharmacists, physical educators, physicians, and physiotherapists) performed data collection. The appraisers were the same in each test. Data collection occurred on three non-consecutive days: 1st) recruitment: inclusion criteria verification by phone calls; 2nd) cognition assessment, anthropometrics, body composition, HGS, mobility and physical activity level assessment; and 3rd) lower limbs muscle strength assessment. These procedures are resumed in Fig. 1.

### Cognition assessment

The validated Mini Mental State Examination (MMSE) was used to assess participants’ cognition status and to ensure that participants understood the other tests executed in the present study [36]. The MMSE was executed in a quiet room, face to face with the researcher. Those who have MMSE ≤ 12 were considered with dementia and were excluded [37].

### Measure of body-size variables

Forty-nine body-size variables (Additional file 1: Supplement A) were collected to propose allometric exponents and to normalize performance in muscle strength tests. The selection of these variables were based on those previously used to calculate body indexes [38–46], and involved anthropometric measurements [47] and body composition (Dual Energy X-ray Absorptiometry-DXA and bioelectrical impedance analysis-BIA), as briefly detailed below (body indexes).

Measures and instruments utilized were: body mass (Filizola® digital scale, model Personal, Brazil), height (Sanny® wall-mounted aluminum stadiometer, Professional model ES2020, Brazil), circumferences (Sanny® inelastic and inextensible measuring tape, Brazil), skinfolds (Lange scientific skinfold caliper, Cambridge Scientific Instruments, Cambridge, Maryland), bone breadths (Sanny® anthropometer and small sliding caliper, Brazil), and segment lengths (Sanny® segmometer, Brazil), lean soft tissue (LST) components, appendicular skeletal muscle mass (ASM) and FFM (DXA, Hologic®, model QDR4500W, software version 11.2, Bedford, MA), FFM [46] (Bioimpedance Imp DF50 Body Composition Analysis, ImpediMed®, Brisbane, Queensland, Australia).

Anthropometry (body mass, height, circumferences, skinfold andbone breadths) was collected according to a standardized procedure published elsewhere [47]. DXA involved a full body scan performed (according to the manufacturer’s recommended procedures) and interpreted always by the same technician. BIA exam was conducted in controlled temperature room (23 ºC) with the older adults backed on a litter in comfortable position after rest for 10 min in supine position, without footwear and adornments (rings and earrings), with legs separated and opened hands. Older adults were previously oriented (24 h before the exam) to avoid the consumption of alcohol and caffeine (coffee, tea, chocolate), diuretic medication, intense physical activity and meal four hours before the exam.

### Body indexes

The body indexes derived from anthropometry were body mass index (BMI, kg/m^2^) [38], body mass*height [39], human body surface area (SA, m^2^) [40], absolute mid-arm muscle circumference (MAMC, cm) [41], corrected arm muscle area (CAMA, cm) [42], arm fat area (AFA,cm^2^) [43], FFM [44] and fat mass (obtained by body mass difference). The body indexes derived from body composition were LST of arms and legs, ASM, ASM/height (m)^2^ [45], FFM estimated from BIA [46] and DXA, when fat mass were estimated by body mass difference.

### Mobility measurement

The cut-off points for muscle weakness were established based on the main outcome (mobility limitation). Mobility was verified based on the six-minute walk test (6MWT) carried out in a corridor 30-m length. Along this path, at every three meters there was a cone to help researcher to precisely identify the walked distance [31]. Participants were instructed to cover the longest distance walking as faster as they could during the six-minute time. Nevertheless, participants could slow down, interrupt the walking, and resume the test whenever desired, although time was not paused. Total walked distance was recorded and mobility limitation was characterized when the 6MWT < 400 m [48].

### Muscle strength measurements

Muscle strength was measured using HGS, one maximum repetition measurement for knee extensors (1RM) and isokinetic knee extension peak torque at a velocity of 60°/s (PT)*.* The maximum HGS was measured with a manual dynamometer (Jamar®, model 5030J1) using a previously published protocol [49]. Three attempts were performed, one minute apart, with the dominant hand and the highest result was recorded in kg as HGS [50, 51]. The 1RM was estimated in a leg extension machine (Lion Fitness® model LFS) with a submaximal repetition protocol: 1RM = weight lifted/(1.0278 − [0.0278*nº of reps]) [52]. The detailed protocol was published elsewhere [26]. Briefly, a warm-up with lowest load was executed with 10 repetitions. After two-min resting, the load was doubled and eight repetitions were performed. After three-min resting, the test started and initial load was based on participants body mass (45% for women and 64% for men). The goal was to perform a maximum of 10 repetitions in three possible attempts (separate with three minutes intervals). Therefore, depending on older adults’ muscle strength level, these initial loads could be increased or decreased to estimate 1RM. The PT of the right lower limb was recorded with the Biodex (model System 4 Pro) isokinetic dynamometer and results are in newton-meter (Nm) according to standardized protocol [53]. Briefly, a warm-up with 10 submaximal repetitions in angular speed of 60°/s was performed. After three-min resting, the test was started with executing five maximum repetitions verbally encouraged by researchers without visual feedback. 1RM was executed prior to the PT, and the time interval between these tests was at least 30 min.

### Physical activity level measurement

The International Physical Activity Questionnaire—Short Version was used to get physical activity level [54]. Physical activity level was dichotomized into sedentary (0) and irregularly active, active or very active (1). These two categories were introduced in the models to provide allometric exponents.

### Muscle strength normalization procedures (ratio standard/muscle quality and allometric scaling)

HGS, 1RM and PT were considered in three different ways: 1) absolute (non-normalized); 2) ratio standard or muscle quality (muscle strength/body-size variable); and 3) allometrically adjusted (muscle strength/body-size variable^b^).

Allometric exponents (^b^) were proposed only for body-size variables that showed significant correlation (Pearson's correlation) with muscle strength. To generate the allometric exponents, muscle strength (Y) and body-size variables (X) were converted to natural logarithm (ln) and the slope of regression line is allometric exponent (^b^), according to more detail previously published [24]. Therefore, allometric exponents were discarded when the interaction (ln body-size variable*age*sex*physical activity level) was significant or when there was multicollinearity in the linear regression (variance inflation factor [VIF] > 10) [55].

We also consider other allometric exponents (^b^) of the literature, as described in Table 1.

**Table 1:** Allometric exponents (^b^) proposed in previous studies

Authors	Normalized muscle strength for body-size variable
Jaric [56]	General muscle strength/body mass^0.67^
Foley et al. [23]	HGS/body mass^0.40^
Pua [25]	HGS/body mass^0.63^
Maranhão Neto et al. [24]	HGS/body mass^0.31^
	HGS/height^1.84^
Abdalla et al. [26]	1RM/body mass^0.69^
	1RM/body mass^0.96^
Davies and Dalsky [28]	PT/body mass^0.67^
	PT/body mass^0.72^
	PT/body mass^0.74^
Segal et al. [39]	PT/body mass*height^0.97^

*HGS* handgrip strength, *1RM* one maximum repetition measurement for knee extensors, *PT* isokinetic knee extension peak torque at 60°/s

In order to verify whether normalization removed the influence of body size on muscle strength, the correlation between normalized muscle strength and body-size variables (body mass, height and body-size used) should be negligible (r ≤ 30) [57].

#### Statistical analysis

We recorded and reviewed the data by double typing, followed by an exploratory analysis for error detection. We use parametric statistics for continuous variables considering the central limit theorem [58].

#### Proposition of cut-off points for muscle weakness

Absolute muscle strength and normalized by ratio standard/muscle quality or allometric scaling had their area under the curve (AUC) quantified by the ROC curve. The Youden index [59] selected the most appropriate cut-off points with the best relationship between sensitivity and specificity for the primary main outcome (functional limitation: 6MWD < 400) [48].

The cut-off points were considered adequate when they have AUC ≥ 0.70 [60] simultaneously for both sexes (*p* < 0.05) and when the correlation between muscle strength and body-size variables (body mass, height and body-size used) were negligible (r ≤ 0.30) [57].

For each muscle strength test (HGS, 1RM and PT), way (non-normalized, ratio standard/muscle quality and allometric scaling) and for each and sex was selected the adequate cut-off point according the superior accuracy. When there was a tie in accuracy, the variable with the greatest sensitivity or specificity was chosen. Finally, the AUC—ROC curves of non-normalized and normalized muscle strength were compared with each other to decide the best cut-off point.

The analyzes were performed using the SPSS 25.0 statistical package, and the ROC curves and Youden index in MedCalc 15.2 with a previously established level of significance (α = 5%).

## Results

Sample was encompassed by 100 older adults (69 women) who agreed to participate in the study. From those, 6 were excluded for different reasons, as the stages of the study proceeded, as detailed in Fig. 1. Therefore, the final sample comprised 29 older men (31%) and 65 older women (69%).

Sample characterization according to sex is shown in Table 2. About main outcome of the study, twenty-five women (38.5%) and seven men (24.1%) had functional limitation (6MWT < 400 m).

**Table 2:** Descriptive analysis and significant correlations of muscle strength with body-size variables in older men and women (n = 94)

Variables	Older Men (n = 29)				Older Women (n = 65)				Correlation (r) with Muscle Strength		
	M	95% CI		SD	M	95% CI		SD	HGS (kg)	Knee Extension	
		LL	UL			LL	UL			1RM (kg)	PT (Nm)
Age (years)	71.2	68.5	73.9	7.1	69.7	68.2	71.2	6.1			
Mini-Mental State Examination (0–19)	17.6	16.9	18.2	1.8	17.4	16.9	17.8	1.8			
*Body-size variables*											
Anthropometry											
Body mass (kg)	73.0	67.7	78.3	13.9	66.9	64.0	69.8	11.6	0.37^†^	0.39^†^	0.40^†^
Height (m)	1.7	1.6	1.7	0.1	1.6	1.5	1.6	0.1	0.71^†^	0.62^†^	0.68^†^
Circumferences (cm)											
Forearm	26.0	25.2	26.8	2.0	23.8	23.3	24.4	2.2	0.50^†^	0.40^†^	0.46^†^
Calf	35.8	34.4	37.1	3.5	34.8	34.1	35.5	2.9	0.34*	0.37^†^	0.37^†^
Chest	97.8	94.3	101.4	9.4	93.2	91.3	95.1	7.7	0.26*	0.32*	0.36^†^
Waist	92.1	87.8	96.5	11.4	86.5	84.0	89.0	10.0		0.26*	0.30*
Skinfold thickness (mm)											
Triceps	15.2	12.9	17.5	6.0	25.8	24.1	27.4	6.7	–0.39^†^	–0.22*	–0.25*
Biceps	8.0	6.7	9.4	3.5	15.4	14.0	16.7	5.4	–0.40^†^	–0.28*	–0.31*
Midaxillary	18.5	15.5	21.5	7.8	23.9	22.2	25.6	6.9	–0.26*		
Pectoral	16.8	14.7	18.9	5.5	14.6	13.0	16.2	6.4	0.21*		
Suprailiac	19.7	15.8	23.5	10.1	29.7	27.8	31.7	7.8	–0.29*		
Abdominal (vertical)	26.2	23.1	29.3	8.1	33.7	31.5	35.9	8.8	–0.22*		
Thigh (midline)	17.9	15.0	20.8	7.6	32.1	29.5	34.8	10.7	–0.35*	–0.27*	–0.38^†^
Medial calf	11.8	9.3	14.3	6.6	23.8	21.9	25.7	7.6	–0.41^†^	–0.28*	–0.33*
Bone breadths (mm)											
Biacromial	39.9	38.9	41.0	2.8	37.1	36.6	37.6	2.1	0.63^†^	0.58^†^	0.60^†^
Bitrochanteric	33.7	33.1	34.3	1.7	33.4	32.8	34.0	2.3		0.20*	0.22*
Ankle (bimalleolar)	7.0	6.8	7.2	0.5	6.3	6.2	6.4	0.4	0.59^†^	0.47^†^	0.52^†^
Elbow	6.7	6.5	6.9	0.5	5.8	5.7	6.0	0.5	0.53^†^	0.40^†^	0.44^†^
Wrist	5.7	5.6	5.9	0.4	5.1	5.0	5.1	0.4	0.52^†^	0.37^†^	0.44^†^
Chest	30.9	29.9	31.9	2.6	27.8	27.4	28.3	1.8	0.58^†^	0.49^†^	0.59^†^
Segment lenghts (cm)											
Knee height	53.5	52.4	54.5	2.7	49.5	49.0	50.0	2.1	0.62^†^	0.55^†^	0.58^†^
Half arm span	87.3	85.5	89.2	4.8	80.8	79.8	81.7	3.8	0.71^†^	0.62^†^	0.58^†^
*Body indexes*											
Derived from anthropometry											
Body mass*height (kg*m)	123.3	112.6	134.1	28.2	104.7	99.8	109.7	20.0	0.49^†^	0.49^†^	0.52^†^
SA (m^2^)	1.9	1.8	1.9	0.2	1.7	1.7	1.8	0.2	0.47^†^	0.48^†^	0.50^†^
MAMC (cm)	24.2	23.0	25.3	2.9	21.9	21.3	22.5	2.6	0.45^†^	0.39^†^	0.45^†^
CAMA (cm^2^)	37.1	32.7	41.6	11.7	32.2	29.9	34.5	9.2	0.37^†^	0.33^†^	0.40^†^
AFA (cm^2^)	16.3	14.0	18.6	6.0	22.9	21.3	24.6	6.6	–0.23*		
FFM_(LEAN et al. [44]__)_ (kg)	52.1	49.6	54.6	6.6	37.1	36.0	38.2	4.6	0.75^†^	0.66^†^	0.67^†^
Fat mass_(LEAN et al. [44])_ (kg)	20.9	17.7	24.0	8.2	29.8	27.9	31.7	7.8	–0.22*		
Derived from body composition											
Left arm LST (kg)	2.4	2.1	2.6	0.6	1.5	1.4	1.6	0.3	0.72^†^	0.62^†^	0.66^†^
Right arm LST (kg)	2.8	2.5	3.0	0.6	1.8	1.7	1.9	0.4	0.72^†^	0.61^†^	0.60^†^
Left leg LST (kg)	7.7	7.1	8.3	1.6	5.5	5.3	5.8	1.0	0.67^†^	0.64^†^	0.64^†^
Right leg LST (kg)	8.0	7.4	8.6	1.6	5.7	5.4	5.9	1.0	0.70^†^	0.64^†^	0.65^†^
Arms LST (kg)	5.1	4.7	5.6	1.2	3.3	3.2	3.5	0.7	0.74^†^	0.63^†^	0.64^†^
Legs LST (kg)	15.7	14.6	16.9	3.1	11.2	10.7	11.6	1.9	0.69^†^	0.65^†^	0.66^†^
ASM (kg)	20.9	19.3	22.5	4.2	14.5	13.9	15.1	2.5	0.72^†^	0.65^†^	0.66^†^
ASM/height^2^ (kg/m^2^)	7.3	7.0	7.7	1.0	6.0	5.7	6.2	0.9	0.55^†^	0.51^†^	0.48^†^
FFM_(Baumgartner et al. [46])_ (kg)	54.3	51.3	57.3	7.7	45.5	44.0	47.0	6.1	0.60^†^	0.55^†^	0.57^†^
FFM_(DXA)_ (kg)	51.5	47.9	55.0	9.4	38.8	37.4	40.3	5.8	0.68^†^	0.59^†^	0.61^†^
Fat mass_(DXA)_ (kg)	21.5	18.8	24.2	7.1	28.1	26.3	29.9	7.2	–0.20*		
*Mobility*											
Six-minute walk test (6MWT)	464.7	431.1	498.3	88.3	412.7	389.9	435.5	92.0			
Functional limitation (6MWT < 400 m); %	24.1%				38.5%						
*Muscle strength*											
HGS (kg)	36.4	33.1	39.7	8.6	24.1	23.0	25.2	4.5			
1RM (kg)	66.8	56.9	76.7	26.0	40.8	36.9	44.8	15.9			
No. of reps to estimate 1RM	7.2	6.3	8.1	2.3	6.6	6.0	7.2	2.4			
PT (Nm)	119.8	102.4	137.2	45.6	73.2	66.8	79.6	25.9			

*M* mean, *CI* confidence interval, *LL* lower limit, *UL* upper limit, *SD* standard deviation, *HGS* handgrip strength, *1RM* one maximum repetition measurement for knee extensors, *PT* isokinetic knee extension peak torque at 60º/s, *Nm* Newton meter, *SA* human body surface area, *MAMC* mid-arm muscle circumference, *CAMA* corrected arm muscle area, *AFA* arm fat area, *FFM* fat-free mass, *LST* lean soft tissue, *ASM* appendicular skeletal muscle mass, *DXA* Dual-energy X-ray absorptiometry

*p < 0.05 and ^†^*p* < 0.001 (statistically significant correlation)

The correlations between muscle strength and body-size variables are also shown in Table 2. Most of the body-size variables showed a significant correlation with muscle strength (r = − 0.41 to 0.75; *p* < 0.05). Non-significant correlations between body-size variables and muscle strength tests are shown in Additional file 1: Supplement B.

Allometric exponents were proposed for those body-size variables that showed a significant relationship with muscle strength (Table 2). Linear regressions to obtain allometric exponents are shown in Additional file 1: Supplement C. All regressions were significant to explain muscle strength (*p* < 0.05), with adjusted R^2^ ranging from 0.39 to 0.61. The regression coefficients (β) obtained for each body-size variable represent the allometric exponents obtained. For HGS, the allometric exponents of triceps, pectoral, abdominal and thigh skinfolds were discarded because the interaction terms were statistically significant (*p* < 0.05) and have accentuated multicollinearity (VIF > 10). The remaining allometric exponents were used to perform normalization (for example, 1RM/body mass^0.44^).

The sex-specific cut-off points proposed for HGS, 1RM and PT (non-normalized, ratio standard/muscle quality and allometric scaling) to identify muscle weakness are presented in the Additional file 1: Supplement D. In the same supplement there are also presented correlations between muscle strength and body size (body mass, height and body-size variable used in normalization).

Non-normalized HGS, 1RM and PT cut-off points to identify muscle weakness were not adequate for both sexes or because they did not present AUC ≥ 0.70 (*p* < 0.05) or because they had a significant association with body size (r > 0.30; *p* < 0.05) (Additional file 1: Supplement D).

Table 3 shows the cut-off points based on the ratio standard/muscle quality and allometric scaling classified as adequate.

**Table 3:** Adequate cut-off points (AUC ≥ 0.70 simultaneously in both sexes and r ≤ 0.30 with body size) of handgrip strength (HGS), one maximum repetition measurement for knee extensors (1RM) and isokinetic knee extension peak torque at 60°/s (PT) to identify muscle weakness

Variable	Unit	Men (n = 29)				Women (n = 65)			
		AUC	Cut-off point (≤)	Sens (%)	Spe (%)	AUC	Cut-off point (≤)	Sens (%)	Spe (%)
***HGS (kg)***									
/Forearm circumference	(cm)	0.74*	1.33	86	59	0.70*	1.04	84	58
***1RM (kg)***									
/Body mass	(kg)	0.77*	0.85	86	68	0.76^†^	0.54	68	78
/Forearm circumference	(cm)	0.75*	2.16	86	77	0.70*	1.38	60	75
/Calf circumference		0.74*	1.65	86	77	0.70*	1.06	72	68
/Chest circumference		0.76*	0.64	86	73	0.71*	0.4	72	65
/Waist circumference		0.78*	0.73	100	59	0.72*	0.37	60	80
/Triceps skinfold	(mm)	0.81^†^	4.22	86	68	0.70*	1.40	60	75
/Bitrochanteric breadth		0.73*	1.72	86	77	0.70*	1.16	76	60
/Bimalleolar breadth		0.73*	8.77	86	77	0.70*	5.77	76	65
/Elbow breadth		0.73*	9.36	86	73	0.70*	6.57	76	63
/SA	(m^2^)	0.75*	31.6	86	77	0.72*	21.2	72	68
/MAMC	(cm)	0.77*	2.42	86	73	0.72*	1.54	64	75
/CAMA	(cm^2^)	0.71*	2.03	100	41	0.73*	0.90	52	93
/FFM_(Lean et al. [44])_	(kg)	0.76*	1.11	86	77	0.72*	1.00	72	68
/FFM_(Baumgartner et al. [46])_	(kg)	0.78*	1.13	83	73	0.75^†^	0.83	76	64
/Body mass^0.44^	(kg)	0.75*	9.04	86	77	0.71*	6.03	76	63
/Body mass^0,67 (Jaric [56])^		0.78*	3.40	86	77	0.73^†^	2.28	76	65
/Body mass^0.96 (Abdalla et al. [53])^		0.77*	1.00	86	68	0.75^†^	0.45	44	100
/Body mass^0.69 (Abdalla et al. [53])^		0.78*	3.06	86	77	0.73*	1.48	44	98
/Calf circumference^1.10^	(cm)	0.75*	1.14	86	77	0.71*	0.70	68	73
/Bimalleolar breadth^1.20^	(mm)	0.71*	6.01	86	68	0.70*	3.93	76	65
/(Body mass*height)^0.48^	(kg*m)	0.75*	5.83	86	77	0.72*	4.06	76	65
/SA^0.93^	(m^2^)	0.75*	33	86	77	0.72*	22.7	76	65
/FFM^0.88^_(Lean et al. [44])_	(kg)	0.75*	1.77	86	77	0.71*	1.53	76	65
/FFM^0.67^_(Baumgartner et al. [46])_		0.76*	3.94	83	77	0.72*	2.91	76	64
***PT*** ***(Nm)***									
/Height	(m)	0.93^†^	54.1	86	95	0.74^†^	44.1	64	75
/Knee height	(cm)	0.94^†^	1.83	100	86	0.75^†^	1.44	68	73
/SA	(m^2^)	0.94^†^	56.9	100	82	0.81^†^	36.3	64	88
/FFM_(Lean et al. [44])_	(kg)	0.93^†^	1.79	86	95	0.81^†^	1.84	76	80
/Left leg LST	(g)	0.82^†^	0.015	100	55	0.76^†^	0.012	68	80
/Right leg LST		0.77*	0.015	100	50	0.78^†^	0.013	76	78
/Legs LST		0.81*	0.0063	71	82	0.77^†^	0.0065	76	78
/ASM	(kg)	0.81*	4.66	71	86	0.77^†^	5.01	76	78
/FFM_(Baumgartner et al. [46])_		0.93^†^	1.63	83	95	0.85^†^	1.60	88	77
/FFM_(DXA)_		0.84^†^	2.08	100	59	0.79^†^	1.89	76	75
/Body mass^0.67 (Davies and Dalsky [28])^	(kg)	0.93^†^	5.06	86	95	0.82^†^	3.71	68	88
/Body mass^0.72 (Davies and Dalsky [28])^		0.94^†^	4.1	86	95	0.82^†^	3.14	72	85
/Body mass^0.74 (Davies and Dalsky [28])^		0.93^†^	3.77	86	95	0.82^†^	2.87	72	85
/Body mass^0.67 (Jaric [56])^		0.93^†^	5.06	86	95	0.82^†^	3.71	68	88
/Height^3.27^	(m)	0.86^†^	19.2	100	77	0.74^†^	17.4	72	65
/Knee height^1.82^	(cm)	0.90^†^	0.068	100	86	0.74^†^	0.053	64	75
/Half arm span^1.62^		0.88^†^	0.076	100	73	0.75^†^	0.066	84	58
/Biacromial breadth^2.15^	(mm)	0.82^†^	0.038	86	68	0.77^†^	0.03	76	73
/Bimalleolar breadth^1.54^		0.81*	5.04	86	73	0.80^†^	4.64	92	60
/(Body mass*height)^0.43^	(kg*m)	0.94^†^	13	100	82	0.80^†^	10.5	84	65
/SA^0.83^	(m^2^)	0.94^†^	53.8	86	95	0.80^†^	50	84	65
/Left leg LST^0.43^	(g)	0.92^†^	2.26	100	77	0.77^†^	1.83	76	70
/Right leg LST^0.48^		0.90^†^	1.39	100	73	0.78^†^	1.16	80	70
/Legs LST^0.47^		0.92^†^	1.07	100	73	0.77^†^	0.87	76	70

Dependent variable (primary outcome): functional limitation (6MWT < 400 m)

*AUC* area under the curve, *p* significance, *Sens* sensibility, *Spe* specificity, *SA* human body surface area, *MAMC* mid-arm muscle circumference, *CAMA* corrected arm muscle area, *FFM* Fat-free mass, *LST* lean soft tissue, *ASM* appendicular skeletal muscle mass, *DXA* Dual-energy X-ray absorptiometry, *6MWT* six-minute walk test

*p < 0.05 and ^†^*p* < 0.001 (statistically significant AUC)

**Fig. 2:**
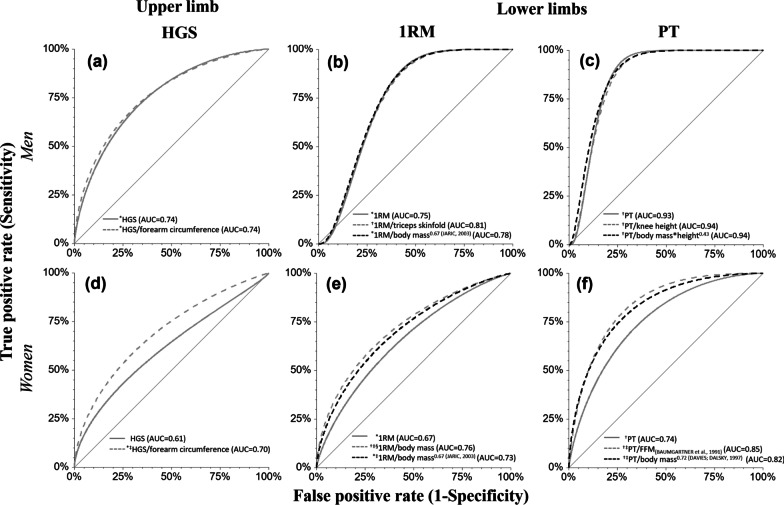
Accuracy comparison between non-normalized, ratio standard/muscle quality and allometric scaling of muscle weakness cut-off points of HGS and lower limbs strength (1RM and PT) in older men (letters a, b, c) and older women (letters d, e, f). *p < 0.05 and ^†^*p* < 0.001 (statistically significant AUC). ^‡^*p* < 0.05 (greater than the AUC of non-normalized muscle strength). ^§^*p* < 0.05 (greater than the AUC of the allometric scaling). Dependent variable (primary outcome): functional limitation (6MWT < 400 m). *HGS* handgrip strength; *1RM* one maximum repetition measurement for knee extensors, *PT* isokinetic knee extension peak torque at 60º/s, *6MWT* six-minute walk test

A comparison of the most accurate ROC curves is presented in Fig. 2 to support the decision for the best cut-off point between non-normalized, ratio standard/muscle quality and allometric scaling of HGS and lower limbs strength (1RM and PT) for each sex.

For men, there were no differences in accuracy (AUC) to identify functional limitation between absolute muscle strength, normalized by ratio standard/muscle quality or by allometric scaling (*p* > 0.05; Fig. 2a–c). However, the absolute muscle strengths (HGS, 1RM and PT) previously indicated great dependence (r > 0.30) on body size (Additional file 1: Supplement D), suggesting the need for normalization to avoid errors in the classification of weakness. The normalized muscle strength increased the AUC and made it possible to classify muscle weakness of older adults with extreme body sizes, independently.

For women, only after normalizing muscle strength the AUC values perform acceptable to identify functional limitation (AUC > 0.70; Fig. 2d, e). The exception was PT, when the absolute values already had adequate accuracy (AUC > 0.70), although without the desirable independence of body size. All the normalizations increased (^‡^) the AUC (*p* < 0.001).

## Discussion

Cut-off points based on upper and lower limbs muscle strength were proposed to identify muscle weakness in older adults of both sexes. The non-normalized cut-off points for HGS and lower limbs strength were significantly associated with body size, which involves biases to assess older adults with extreme body size (e.g., heavy or short). After normalizing HGS and lower limbs strength by the ratio standard/muscle quality or by the allometry, the association with body size was no longer relevant. In addition, for women, the accuracy to predict mobility limitation/muscle weakness from normalized muscle strength cut-off points become acceptable when compared to non-normalized strategy. In men, muscle strength normalization did not increase accuracy. However, all normalized models of both sexes avoided biases in the assessment of muscle weakness/mobility limitation, to isolate the natural interdependence between muscle strength and body size [24].

To the best of our knowledge, this is the first study to propose muscle weakness cut-off points for the HGS and PT allometrically adjusted in older adults. In a previous study, 1RM was allometrically adjusted for body mass [26], but not according to all other potential body size variables. Indeed, we expanded the number of variables that can be used to normalize 1RM with allometry (n = 8) in order to augment model´s accuracy for identifying muscle weakness regardless of extreme body sizes. Other studies proposed muscle weakness cut-off points with HGS normalized by ratio standard (body mass or BMI) [18, 61, 62] or stratified by BMI quartiles [7]. There are also muscle weakness cut-off points for PT normalized by body mass [19]. However, these studies did not compare the accuracy of normalized with non-normalized muscle strength to identify muscle weakness. Furthermore, they did not explore other body-size variables to normalize muscle strength.

Previous studies have proposed allometric exponents to normalize muscle strength, including HGS [23–25], 1RM [26], PT [28, 39], and they are comparable with the ones found in the present study. Curvilinear (allometric) relationship variables is confirmed when allometric coefficient (^b^) is between 0.00 and 0.99 [63], while the linear relationship is characterized when the exponent is ≥ 1.00 [63]. In the literature, body mass generally presents an allometric relationship with muscle strength independently of the test (HGS, 1RM or PT; Table 1), confirming our findings (Additional file 1: Supplement C), when ^b^ exponents were 0.22 (HGS), 0.44 (1RM) and 0.37 (PT). Contrarily, height tends to have a linear relationship (^b^ ≥ 1.00) with muscle strength [24], what was also confirmed by our proposed allometric exponents (Additional file 1: Supplement C), that were between 1.87 and 3.27.

Some strengths of our study are noteworthy. We proposed muscle weakness cut-off points for isokinetic dynamometer, considered as a “gold standard” resource to assess lower limbs strength. The estimated 1RM obtained with submaximal repetition protocol and the HGS are valid for older adults, even for those with muscle weakness [53, 64]. An extensive number of body-size variables (n = 49) were tested in our study, expanding the possibilities to promote the normalization of performance in muscle strength tests. Furthermore, regardless of the chosen muscle strength test to evaluate weakness, our findings can be applied with sufficient accuracy (AUC > 0.70) both for scientific research (PT) and population-based monitoring (HGS and 1RM). Nevertheless, this study is not without limitations. The individual muscle strength decline along aging may have been underestimated with the cross-sectional design. The small and local sample size of our study, requiring caution to extrapolate these findings inferentially to other populations. Another limitation is the utilization of open kinetic chain test in the case of 1RM in a leg extension machine, a movement far to the natural comportment during daily living. Our suggestion for future studies is to establish allometric exponents and cut-off points for a close kinetic chain exercise like leg press or squat, which require movements more closely associated with daily live.

We found greater accuracy (AUC) for normalized lower limbs strength (isokinetic dynamometer and leg extension machine) than manual dynamometer (normalized upper extremity strength), usually adopted to predict mobility limitations/muscle weakness [8]. However, the isokinetic dynamometer is expensive and generally more available in terms of research. Even though, our normalized models are also applicable in clinical practice from manual dynamometers (widely available in geriatric environments) and leg extension machines (available in most fitness centers, adequate environment for intervention against aged-related muscle weakness) [35]. The assessment of HGS and 1RM and proper classification of muscle weakness amongst older adults should be frequent in clinical practice to better target health expending, avoiding unnecessary expenditures. Future research should observe if proposed allometric exponents can be utilized to normalize muscle strength for different older adults’ population, with other ethnicity/race characteristics.

As an applied example to avoid false positive diagnosis for muscle weakness, we hypothesize one older man with extreme lower values of body mass (42 kg), 1.57 m of height, who performed PT of 85.2 Nm. If we consider our absolute cut-off point (≤ 85.4 Nm), this older man has muscle weakness confirmed. However, when considered the normalized PT/([body mass*height]^0.43^), the adjusted value (14.1 Nm/kg*m) is above of the cut-off point (13.0 Nm/kg*m; Table 3). Normalization would also avoid false negative cases, for large body size of older adults. For example, if an older woman with 90 kg performs 1RM of 38.2 kg and considering our absolute cut-off point (≤ 38.1 kg), this older woman does not have weakness. However, when considered the normalized (1RM/body mass^0.67^), the adjusted value (1.87 kg/kg) is below of the cut-off point (2.28 kg/kg; Table 3), characterizing weakness and a false negative case if non-normalized cut-off point were considered. The mistaken framing of false weakness cases could greatly impact the financial resources in the health and older people care systems. Especially in low- and middle-income countries, where these resources are scarcer.

## Conclusion

Upper and lower limbs muscle weakness cut-off points standardized according to body size were proposed for older adults of both sexes. The normalization has increased accuracy for identify women with muscle weakness; but not in men, whose absolute muscle strength values have an acceptable accuracy. However, normalization made muscle strength independent of body size, confirming our hypothesis and preventing bias in the evaluation of older adults with extreme body size (e.g., very low or very heavy). Forty-nine valid models were proposed for older adults of both sexes, with different possibilities of body’s normalization of muscle strength, which broadens the interpretation of muscle strength with less risk of attributing a false-negative/positive diagnosis to muscle weakness.

## Supplementary Information

**Additional file 1**. **SUPPLEMENT A** - Body size variables (n = 49) to normalize muscle strength. **SUPPLEMENT B** - Non-significant correlations between body-size variables and muscle strength tests. **SUPPLEMENT C** - Linear regressions to obtain allometric exponents for handgrip strength (HGS), one maximum repetition measurement for knee extensors (1RM) and isokinetic knee extension peak torque at 60º/s (PT) in older men and women (n = 94). **SUPPLEMENT D** - Cut-off points to identify muscle weakness in older adults of the handgrip strength (HGS), one maximum repetition measurement for knee extensors (1RM) and isokinetic knee extension peak torque at 60º/s (PT) (non-normalized, ratio standard/muscle quality and allometric scaling), and the correlation of muscle strength with body size.

## Data Availability

The datasets generated during and/or analysed during the current study are available from the corresponding author on reasonable request.

## Abbreviations

^b^: Allometric exponent
HGS: Handgrip strength
PT: Isokinetic knee extension peak torque at 60°/s
1RM: One maximum repetition measurement for knee extensors
6MWT: Six-minute walk test
