# Supplementary material for: Identification of muscle weakness in older adults from normalized upper and lower limbs strength: a cross-sectional study
Source: BMC Sports Sci Med Rehabil. 2021 Dec 18;13:161. doi: 10.1186/s13102-021-00390-1 (PMC8684151; doi:10.1186/s13102-021-00390-1)
Supplement: Supplementary file 1 — Additional file 1. SUPPLEMENT A - Body size variables (n = 49) to normalize muscle strength. SUPPLEMENT B - Non-significant correlations between body-size variables and muscle strength tests. SUPPLEMENT C - Linear regressions to obtain allometric exponents for handgrip strength (HGS), one maximum repetition measurement for knee extensors (1RM) and isokinetic knee extension peak torque at 60º/s (PT) in older men and women (n = 94). SUPPLEMENT D - Cut-off points to identify muscle weakness in older adults of the handgrip strength (HGS), one maximum repetition measurement for knee extensors (1RM) and isokinetic knee extension peak torque at 60º/s (PT) (non-normalized, ratio standard/muscle quality and allometric scaling), and the correlation of muscle strength with body size. [file 13102_2021_390_MOESM1_ESM.docx]

**ADDITIONAL FILE 1**

SUPPLEMENT A - Body size variables (n=49) to normalize muscle strength.

| **Anthropometry** | | | | |
| --- | --- | --- | --- | --- |
| Body mass (kg) | | | | |
| Height (m) | | | | |
|  | | | | |
| *Circumferences (cm)* | *Skinfold site (mm)* | | *Bone breadths (mm)* |  |
| Arm | Subescapular | | Biacromial |  |
| Forearm | Triceps | | Biiliac |  |
| Midthigh | Biceps | | Bitrochanteric |  |
| Calf | Midaxillary | | Ankle (bimalleolar) |  |
| Chest | Pectoral (chest) | | Elbow |  |
| Waist | Suprailiac | | Wrist |  |
| Abdomen | Abdominal (vertical) | | Knee |  |
| Buttocks (hip) | Thigh (midline) | | Chest |  |
|  | Medial calf | |  |  |
| *Segment lenghts (cm)* |  | |  |  |
| Knee height |  | |  |  |
| Half arm span |  | |  |  |
|  |  | |  |  |
| **Body indexes** | | | | |
| *Derived from anthropometry* | | *Derived from body composition* | |  |
| BMI (kg/m²) | | Left arm LST (g) | |  |
| Body mass*height (kg*m) | | Right arm LST (g) | |  |
| SA (m²) | | Left leg LST (g) | |  |
| MAMC (cm) | | Right leg LST (g) | |  |
| CAMA (cm²) | | Arms LST (g) | |  |
| AFA (cm²) | | Legs LST (g) | |  |
| FFM_(LEAN et al., 1996)_ (kg) | | ASM (kg) | |  |
| Fat mass_(LEAN et al., 1996)_ (kg) | | ASM/height² (kg/m²) | |  |
|  | | FFM_(BAUMGARTNER et al., 1991)_ (kg)  FFM_(DXA)_ (kg) | |  |
|  | | Fat mass_(BAUMGARTNER et al., 1991)_ (kg)  Fat mass_(DXA)_ (kg) | |  |

*Note*: BMI=body mass index; SA=surface area of human body; MAMC=mid-arm muscle circumference; CAMA=corrected arm muscle area; AFA=arm fat area; FFM=fat-free mass; LST=lean soft tissue; ASM=appendicular skeletal muscle mass.

.

SUPPLEMENT B - Non-significant correlations between body-size variables and muscle strength tests

| **Variables** | **Correlation (r) with Muscle Strength** | | |
| --- | --- | --- | --- |
|  | HGS (kg) | Knee Extension | |
|  |  | 1RM (kg) | PT (Nm) |
| ***Body-size variables*** |  |  |  |
| **Anthropometry** |  |  |  |
| *Circumferences (cm)* |  |  |  |
| Arm | 0.08 | 0.15 | 0.18 |
| Midthigh | -0.07 | 0.05 | 0.03 |
| Abdomen | 0.08 | 0.13 | 0.13 |
| Buttocks (hip) | -0.02 | 0.01 | 0.01 |
| *Skinfold thickness (mm)* |  |  |  |
| Subescapular | -0.16 | 0.04 | 0.02 |
| Midaxillary | -0.26^*^ | -0.08 | -0.08 |
| Suprailiac | -0.29^*^ | -0.15 | -0.14 |
| Abdominal (vertical) | -0.22^*^ | -0.14 | -0.11 |
| *Bone breadths (mm)* |  |  |  |
| Knee | 0.14 | 0.16 | 0.16 |
| **Body indexes** |  |  |  |
| *Derived from anthropometry* |  |  |  |
| BMI (kg/m²) | -0.06 | 0.02 | -0.02 |
| AFA (cm²) | -0.23^*^ | -0.08 | -0.08 |
| Fat mass_(LEAN et al., 1996)_ (kg) | -0.22^*^ | -0.09 | -0.09 |
| *Derived from body composition* |  |  |  |
| Fat mass_(BAUMGARTNER et al., 1991)_ (kg) | 0.01 | 0.10 | 0.10 |
| Fat mass_(DXA)_ (kg) | -0.20^*^ | -0.06 | -0.07 |

*Note*: HGS=handgrip strength; 1RM=one maximum repetition measurement for knee extensors; PT=isokinetic knee extension peak torque at 60º/s; Nm=Newton meter; AFA=arm fat area; DXA=Dual-energy X-ray absorptiometry.

SUPPLEMENT C - Linear regressions to obtain allometric exponents for handgrip strength (HGS), one maximum repetition measurement for knee extensors (1RM) and isokinetic knee extension peak torque at 60º/s (PT) in older men and women (n=94).

| **Linear regression** | **Independent variables** | **Regression coefficients** | |  | **95% CI of β** | | **VIF** | **Adjusted R²** |
| --- | --- | --- | --- | --- | --- | --- | --- | --- |
|  |  | **β** | **Standard error** |  | **LL** | **UL** |  |  |
|  |  |  |  |  |  |  |  |  |
| ***Dependent variable: HGS (ln kg)*** | | | | | | | | |
| 1 | Constant | 3.040^†^ | 0.669 |  | 1.711 | 4.368 |  | 0.53 |
|  | Sex | 0.407^†^ | 0.046 |  | 0.315 | 0.499 | 1.129 |  |
|  | Age | -0.012^*^ | 0.003 |  | -0.019 | -0.005 | 1.181 |  |
|  | Physical activity level | 0.061 | 0.144 |  | -0.225 | 0.347 | 1.059 |  |
|  | ln body mass (kg) | 0.219 | 0.122 |  | -0.024 | 0.462 | 1.218 |  |
| 2 | Constant | 3.069^†^ | 0.380 |  | 2.314 | 3.823 |  | 0.58 |
|  | Sex | 0.282^†^ | 0.057 |  | 0.169 | 0.395 | 1.919 |  |
|  | Age | -0.010^*^ | 0.003 |  | -0.017 | -0.004 | 1.143 |  |
|  | Physical activity level | -0.009 | 0.136 |  | -0.279 | 0.262 | 1.068 |  |
|  | ln height (m) | 1.875^†^ | 0.491 |  | 0.899 | 2.85 | 1.891 |  |
| 3 | Constant | 2.393^*^ | 0.877 |  | 0.651 | 4.135 |  | 0.54 |
|  | Sex | 0.381^†^ | 0.050 |  | 0.281 | 0.480 | 1.334 |  |
|  | Age | -0.012^†^ | 0.003 |  | -0.019 | -0.006 | 1.128 |  |
|  | Physical activity level | 0.044 | 0.143 |  | -0.239 | 0.328 | 1.056 |  |
|  | ln forearm circumference (cm) | 0.501^*^ | 0.240 |  | 0.023 | 0.978 | 1.349 |  |
| 4 | Constant | 1.992 | 1.002 |  | <0.001 | 3.984 |  | 0.54 |
|  | Sex | 0.413^†^ | 0.045 |  | 0.323 | 0.502 | 1.084 |  |
|  | Age | -0.012^*^ | 0.003 |  | -0.018 | -0.005 | 1.186 |  |
|  | Physical activity level | 0.084 | 0.143 |  | -0.200 | 0.368 | 1.071 |  |
|  | ln calf circumference (cm) | 0.536^*^ | 0.242 |  | 0.056 | 1.017 | 1.195 |  |
| 5 | Constant | 3.728^*^ | 1.227 |  | 1.289 | 6.167 |  | 0.52 |
|  | Sex | 0.424^†^ | 0.047 |  | 0.329 | 0.518 | 1.143 |  |
|  | Age | -0.014^†^ | 0.003 |  | -0.021 | -0.007 | 1.110 |  |
|  | Physical activity level | 0.048 | 0.146 |  | -0.242 | 0.338 | 1.057 |  |
|  | ln chest circumference (cm) | 0.084 | 0.248 |  | -0.409 | 0.576 | 1.162 |  |
| 6 | Constant | -1.116 | 1.815 |  | -4.722 | 2.489 |  | 0.56 |
|  | Sex | 0.323^†^ | 0.057 |  | 0.210 | 0.435 | 1.779 |  |
|  | Age | -0.013^†^ | 0.003 |  | -0.019 | -0.006 | 1.061 |  |
|  | Physical activity level | 0.02 | 0.140 |  | -0.258 | 0.297 | 1.061 |  |
|  | ln knee height (cm) | 1.318^*^ | 0.451 |  | 0.422 | 2.213 | 1.708 |  |
| 7 | Constant | -4.217^*^ | 1.867 |  | -7.927 | -0.507 |  | 0.61 |
|  | Sex | 0.283^†^ | 0.052 |  | 0.179 | 0.387 | 1.705 |  |
|  | Age | -0.010^*^ | 0.003 |  | -0.016 | -0.003 | 1.161 |  |
|  | Physical activity level | 0.086 | 0.132 |  | -0.176 | 0.349 | 1.061 |  |
|  | ln half arm span (cm) | 1.813^†^ | 0.402 |  | 1.015 | 2.611 | 1.752 |  |
| 8 | Constant | 2.942^†^ | 0.659 |  | 1.633 | 4.251 |  | 0.54 |
|  | Sex | 1.070^*^ | 0.298 |  | 0.478 | 1.663 | 47.067 |  |
|  | Age | -0.011^*^ | 0.004 |  | -0.018 | -0.004 | 1.279 |  |
|  | Physical activity level | 0.647^*^ | 0.308 |  | 0.035 | 1.258 | 4.896 |  |
|  | ln triceps skinfold (mm) | 0.114 | 0.079 |  | -0.043 | 0.270 | 3.147 |  |
|  | Interaction | -0.003^*^ | 0.001 |  | -0.006 | <0.001 | 36.925 |  |
| 9 | Constant | 4.213^†^ | 0.343 |  | 3.530 | 4.895 |  | 0.52 |
|  | Sex | 0.413^†^ | 0.059 |  | 0.296 | 0.53 | 1.752 |  |
|  | Age | -0.015^†^ | 0.003 |  | -0.021 | -0.008 | 1.041 |  |
|  | Physical activity level | 0.037 | 0.148 |  | -0.257 | 0.331 | 1.086 |  |
|  | ln biceps skinfold (mm) | -0.021 | 0.053 |  | -0.126 | 0.083 | 1.706 |  |
| 10 | Constant | 4.160^†^ | 0.343 |  | 3.478 | 4.843 |  | 0.52 |
|  | Sex | 0.425^†^ | 0.049 |  | 0.329 | 0.522 | 1.197 |  |
|  | Age | -0.015^†^ | 0.003 |  | -0.021 | -0.008 | 1.035 |  |
|  | Physical activity level | 0.044 | 0.147 |  | -0.247 | 0.336 | 1.067 |  |
|  | ln midaxillary skinfold (mm) | -0.007 | 0.050 |  | -0.106 | 0.091 | 1.158 |  |
| 11 | Constant | 2.809^†^ | 0.523 |  | 1.769 | 3.848 |  | 0.56 |
|  | Sex | 1.055^†^ | 0.256 |  | 0.546 | 1.564 | 36.193 |  |
|  | Age | -0.011^*^ | 0.003 |  | -0.018 | -0.005 | 1.154 |  |
|  | Physical activity level | 0.769^*^ | 0.307 |  | 0.160 | 1.378 | 5.064 |  |
|  | ln pectoral skinfold (mm) | 0.141^*^ | 0.055 |  | 0.032 | 0.249 | 1.384 |  |
|  | Interaction | -0.003^*^ | 0.001 |  | -0.006 | -0.001 | 36.287 |  |
| 12 | Constant | 4.098^†^ | 0.370 |  | 3.363 | 4.833 |  | 0.52 |
|  | Sex | 0.431^†^ | 0.053 |  | 0.327 | 0.536 | 1.412 |  |
|  | Age | -0.014^†^ | 0.003 |  | -0.021 | -0.008 | 1.085 |  |
|  | Physical activity level | 0.049 | 0.147 |  | -0.243 | 0.341 | 1.069 |  |
|  | ln suprailiac skinfold (mm) | 0.007 | 0.050 |  | -0.093 | 0.106 | 1.432 |  |
| 13 | Constant | 2.458^*^ | 0.682 |  | 1.101 | 3.814 |  | 0.55 |
|  | Sex | 1.253^†^ | 0.312 |  | 0.632 | 1.874 | 53.064 |  |
|  | Age | -0.010^*^ | 0.004 |  | -0.017 | -0.003 | 1.302 |  |
|  | Physical activity level | 0.891^*^ | 0.346 |  | 0.203 | 1.578 | 6.352 |  |
|  | ln abdominal skinfold (mm) | 0.148^*^ | 0.071 |  | 0.008 | 0.289 | 1.86 |  |
|  | Interaction | -0.004^*^ | 0.001 |  | -0.006 | -0.001 | 47.986 |  |
| 14 | Constant | 2.951^†^ | 0.619 |  | 1.720 | 4.181 |  | 0.54 |
|  | Sex | 1.059^†^ | 0.284 |  | 0.495 | 1.624 | 42.851 |  |
|  | Age | -0.011^*^ | 0.004 |  | -0.018 | -0.004 | 1.246 |  |
|  | Physical activity level | 0.676^*^ | 0.314 |  | 0.052 | 1.300 | 5.107 |  |
|  | ln thigh skinfold (mm) | 0.092 | 0.064 |  | -0.034 | 0.219 | 2.412 |  |
|  | Interaction | -0.003^*^ | 0.001 |  | -0.006 | <0.001 | 35.064 |  |
| 15 | Constant | 4.256^†^ | 0.335 |  | 3.590 | 4.921 |  | 0.52 |
|  | Sex | 0.403^†^ | 0.058 |  | 0.287 | 0.519 | 1.737 |  |
|  | Age | -0.015^†^ | 0.003 |  | -0.021 | -0.008 | 1.041 |  |
|  | Physical activity level | 0.041 | 0.146 |  | -0.249 | 0.331 | 1.06 |  |
|  | ln medial calf skinfold (mm) | -0.031 | 0.046 |  | -0.121 | 0.060 | 1.711 |  |
| 16 | Constant | -0.060 | 1.519 |  | -3.077 | 2.958 |  | 0.56 |
|  | Sex | 0.348^†^ | 0.052 |  | 0.244 | 0.452 | 1.506 |  |
|  | Age | -0.010^*^ | 0.004 |  | -0.017 | -0.003 | 1.295 |  |
|  | Physical activity level | 0.091 | 0.141 |  | -0.189 | 0.371 | 1.07 |  |
|  | ln biacromial breadth (mm) | 1.059^*^ | 0.378 |  | 0.308 | 1.810 | 1.689 |  |
| 17 | Constant | 0.536 | 1.234 |  | -1.917 | 2.988 |  | 0.56 |
|  | Sex | 0.414^†^ | 0.044 |  | 0.327 | 0.501 | 1.072 |  |
|  | Age | -0.012^†^ | 0.003 |  | -0.018 | -0.005 | 1.101 |  |
|  | Physical activity level | 0.053 | 0.139 |  | -0.224 | 0.329 | 1.056 |  |
|  | ln bitrochanteric breadth (mm) | 0.971^*^ | 0.325 |  | 0.324 | 1.617 | 1.091 |  |
| 18 | Constant | 2.339^*^ | 0.669 |  | 1.009 | 3.668 |  | 0.56 |
|  | Sex | 0.337^†^ | 0.054 |  | 0.231 | 0.444 | 1.593 |  |
|  | Age | -0.013^†^ | 0.003 |  | -0.019 | -0.007 | 1.043 |  |
|  | Physical activity level | 0.050 | 0.139 |  | -0.228 | 0.327 | 1.056 |  |
|  | ln bimalleolar breadth (mm) | 0.915^*^ | 0.313 |  | 0.292 | 1.537 | 1.538 |  |
| 19 | Constant | 3.443^†^ | 0.565 |  | 2.32 | 4.566 |  | 0.53 |
|  | Sex | 0.375^†^ | 0.059 |  | 0.259 | 0.492 | 1.792 |  |
|  | Age | -0.014^†^ | 0.003 |  | -0.020 | -0.007 | 1.037 |  |
|  | Physical activity level | 0.029 | 0.145 |  | -0.260 | 0.317 | 1.065 |  |
|  | ln elbow breadth (mm) | 0.375 | 0.269 |  | -0.159 | 0.908 | 1.704 |  |
| 20 | Constant | 3.190^†^ | 0.528 |  | 2.141 | 4.239 |  | 0.54 |
|  | Sex | 0.355^†^ | 0.057 |  | 0.242 | 0.468 | 1.715 |  |
|  | Age | -0.014^†^ | 0.003 |  | -0.021 | -0.008 | 1.018 |  |
|  | Physical activity level | 0.036 | 0.143 |  | -0.248 | 0.319 | 1.058 |  |
|  | ln wrist breadth (mm) | 0.576^*^ | 0.277 |  | 0.026 | 1.125 | 1.638 |  |
| 21 | Constant | 2.929^*^ | 0.855 |  | 1.230 | 4.628 |  | 0.53 |
|  | Sex | 0.384^†^ | 0.054 |  | 0.278 | 0.491 | 1.504 |  |
|  | Age | -0.013^†^ | 0.003 |  | -0.02 | -0.007 | 1.068 |  |
|  | Physical activity level | 0.049 | 0.144 |  | -0.238 | 0.336 | 1.056 |  |
|  | ln chest breadth (mm) | 0.338 | 0.228 |  | -0.114 | 0.791 | 1.468 |  |
| 22 | Constant | 2.709^†^ | 0.656 |  | 1.407 | 4.012 |  | 0.55 |
|  | Sex | 0.384^†^ | 0.048 |  | 0.289 | 0.479 | 1.243 |  |
|  | Age | -0.011^*^ | 0.003 |  | -0.018 | -0.004 | 1.207 |  |
|  | Physical activity level | 0.056 | 0.142 |  | -0.226 | 0.337 | 1.057 |  |
|  | ln body mass*height (kg*m) | 0.256^*^ | 0.108 |  | 0.042 | 0.470 | 1.339 |  |
| 23 | Constant | 3.641^†^ | 0.345 |  | 2.955 | 4.327 |  | 0.54 |
|  | Sex | 0.388^†^ | 0.048 |  | 0.293 | 0.483 | 1.222 |  |
|  | Age | -0.011^*^ | 0.003 |  | -0.018 | -0.005 | 1.203 |  |
|  | Physical activity level | 0.057 | 0.142 |  | -0.225 | 0.339 | 1.057 |  |
|  | ln SA (m²) | 0.489^*^ | 0.214 |  | 0.064 | 0.915 | 1.317 |  |
| 24 | Constant | 3.417^†^ | 0.696 |  | 2.035 | 4.799 |  | 0.52 |
|  | Sex | 0.405^†^ | 0.050 |  | 0.306 | 0.504 | 1.276 |  |
|  | Age | -0.013^†^ | 0.003 |  | -0.020 | -0.007 | 1.129 |  |
|  | Physical activity level | 0.034 | 0.145 |  | -0.255 | 0.323 | 1.062 |  |
|  | ln MAMC (cm) | 0.208 | 0.186 |  | -0.162 | 0.579 | 1.292 |  |
| 25 | Constant | 3.773^†^ | 0.427 |  | 2.925 | 4.622 |  | 0.52 |
|  | Sex | 0.414^†^ | 0.047 |  | 0.321 | 0.508 | 1.141 |  |
|  | Age | -0.013^†^ | 0.003 |  | -0.020 | -0.007 | 1.134 |  |
|  | Physical activity level | 0.034 | 0.145 |  | -0.255 | 0.323 | 1.063 |  |
|  | ln CAMA (cm²) | 0.083 | 0.076 |  | -0.067 | 0.233 | 1.180 |  |
| 26 | Constant | 4.087^†^ | 0.375 |  | 3.342 | 4.832 |  | 0.52 |
|  | Sex | 0.432^†^ | 0.051 |  | 0.331 | 0.532 | 1.299 |  |
|  | Age | -0.014^†^ | 0.003 |  | -0.021 | -0.008 | 1.105 |  |
|  | Physical activity level | 0.047 | 0.146 |  | -0.243 | 0.337 | 1.057 |  |
|  | ln AFA (cm²) | 0.010 | 0.058 |  | -0.106 | 0.126 | 1.353 |  |
| 27 | Constant | 1.747 | 0.897 |  | -0.036 | 3.530 |  | 0.56 |
|  | Sex | 0.242^*^ | 0.080 |  | 0.083 | 0.401 | 3.528 |  |
|  | Age | -0.008^*^ | 0.004 |  | -0.016 | -0.001 | 1.529 |  |
|  | Physical activity level | 0.086 | 0.141 |  | -0.194 | 0.366 | 1.067 |  |
|  | ln FFM_(LEAN et al., 1996)_ (kg) | 0.531^*^ | 0.191 |  | 0.152 | 0.910 | 3.814 |  |
| 28 | Constant | 3.934^†^ | 0.391 |  | 3.157 | 4.711 |  | 0.52 |
|  | Sex | 0.447^†^ | 0.053 |  | 0.342 | 0.551 | 1.414 |  |
|  | Age | -0.014^†^ | 0.003 |  | -0.021 | -0.008 | 1.059 |  |
|  | Physical activity level | 0.050 | 0.146 |  | -0.239 | 0.339 | 1.057 |  |
|  | ln fat mass_(LEAN et al., 1996)_ (kg) | 0.048 | 0.067 |  | -0.085 | 0.180 | 1.415 |  |
| 29 | Constant | 1.774^*^ | 0.861 |  | 0.063 | 3.485 |  | 0.56 |
|  | Sex | 0.298^†^ | 0.063 |  | 0.174 | 0.423 | 2.181 |  |
|  | Age | -0.011^*^ | 0.003 |  | -0.018 | -0.004 | 1.174 |  |
|  | Physical activity level | 0.036 | 0.140 |  | -0.241 | 0.314 | 1.057 |  |
|  | ln left arm LST (g) | 0.290^*^ | 0.101 |  | 0.090 | 0.490 | 2.182 |  |
| 30 | Constant | 1.864^*^ | 0.917 |  | 0.042 | 3.685 |  | 0.55 |
|  | Sex | 0.310^†^ | 0.063 |  | 0.184 | 0.436 | 2.187 |  |
|  | Age | -0.011^*^ | 0.003 |  | -0.018 | -0.004 | 1.258 |  |
|  | Physical activity level | 0.065 | 0.141 |  | -0.215 | 0.345 | 1.059 |  |
|  | ln right arm LST (g) | 0.264^*^ | 0.102 |  | 0.061 | 0.467 | 2.282 |  |
| 31 | Constant | 1.314 | 1.213 |  | -1.096 | 3.723 |  | 0.55 |
|  | Sex | 0.326^†^ | 0.062 |  | 0.203 | 0.448 | 2.049 |  |
|  | Age | -0.011^*^ | 0.004 |  | -0.018 | -0.004 | 1.275 |  |
|  | Physical activity level | 0.053 | 0.142 |  | -0.228 | 0.334 | 1.056 |  |
|  | ln left leg LST (g) | 0.296^*^ | 0.124 |  | 0.049 | 0.543 | 2.149 |  |
| 32 | Constant | 1.202 | 1.271 |  | -1.324 | 3.728 |  | 0.54 |
|  | Sex | 0.319^†^ | 0.064 |  | 0.192 | 0.446 | 2.211 |  |
|  | Age | -0.011^*^ | 0.004 |  | -0.018 | -0.004 | 1.288 |  |
|  | Physical activity level | 0.077 | 0.142 |  | -0.205 | 0.360 | 1.065 |  |
|  | ln right leg LST (g) | 0.305^*^ | 0.129 |  | 0.048 | 0.562 | 2.351 |  |
| 33 | Constant | 1.418 | 0.995 |  | -0.559 | 3.394 |  | 0.56 |
|  | Sex | 0.295^†^ | 0.064 |  | 0.167 | 0.422 | 2.287 |  |
|  | Age | -0.010^*^ | 0.003 |  | -0.017 | -0.004 | 1.237 |  |
|  | Physical activity level | 0.053 | 0.140 |  | -0.225 | 0.331 | 1.056 |  |
|  | ln arms LST (g) | 0.299^*^ | 0.106 |  | 0.089 | 0.509 | 2.346 |  |
| 34 | Constant | 0.879 | 1.365 |  | -1.835 | 3.592 |  | 0.55 |
|  | Sex | 0.317^†^ | 0.064 |  | 0.190 | 0.443 | 2.194 |  |
|  | Age | -0.011^*^ | 0.004 |  | -0.018 | -0.003 | 1.298 |  |
|  | Physical activity level | 0.067 | 0.142 |  | -0.215 | 0.348 | 1.06 |  |
|  | ln legs LST (g) | 0.317^*^ | 0.130 |  | 0.058 | 0.576 | 2.324 |  |
| 35 | Constant | 2.917^†^ | 0.534 |  | 1.856 | 3.979 |  | 0.55 |
|  | Sex | 0.303^†^ | 0.065 |  | 0.174 | 0.432 | 2.299 |  |
|  | Age | -0.010^*^ | 0.004 |  | -0.017 | -0.003 | 1.302 |  |
|  | Physical activity level | 0.064 | 0.141 |  | -0.216 | 0.344 | 1.058 |  |
|  | ln ASM (kg) | 0.335^*^ | 0.127 |  | 0.082 | 0.588 | 2.422 |  |
| 36 | Constant | 3.808^†^ | 0.463 |  | 2.888 | 4.729 |  | 0.52 |
|  | Sex | 0.400^†^ | 0.056 |  | 0.288 | 0.511 | 1.601 |  |
|  | Age | -0.013^†^ | 0.003 |  | -0.02 | -0.007 | 1.179 |  |
|  | Physical activity level | 0.061 | 0.146 |  | -0.23 | 0.352 | 1.070 |  |
|  | ln ASM/height² (kg/m²) | 0.130 | 0.149 |  | -0.167 | 0.426 | 1.685 |  |
| 37 | Constant | 2.112^*^ | 0.764 |  | 0.593 | 3.631 |  | 0.58 |
|  | Sex | 0.375^†^ | 0.051 |  | 0.273 | 0.477 | 1.503 |  |
|  | Age | -0.009^*^ | 0.003 |  | -0.016 | -0.002 | 1.235 |  |
|  | Physical activity level | 0.114 | 0.137 |  | -0.159 | 0.386 | 1.074 |  |
|  | ln FFM_(BAUMGARTNER et al., 1991)_ (kg) | 0.412^*^ | 0.157 |  | 0.099 | 0.724 | 1.665 |  |
| 38 | Constant | 2.569^†^ | 0.680 |  | 1.218 | 3.920 |  | 0.55 |
|  | Sex | 0.325^†^ | 0.060 |  | 0.205 | 0.445 | 1.979 |  |
|  | Age | -0.011^*^ | 0.003 |  | -0.018 | -0.004 | 1.231 |  |
|  | Physical activity level | 0.059 | 0.141 |  | -0.222 | 0.339 | 1.057 |  |
|  | ln FFM_(DXA)_ (kg) | 0.356^*^ | 0.142 |  | 0.073 | 0.638 | 2.054 |  |
| 39 | Constant | 3.988^†^ | 0.408 |  | 3.177 | 4.799 |  | 0.52 |
|  | Sex | 0.438^†^ | 0.050 |  | 0.338 | 0.537 | 1.268 |  |
|  | Age | -0.014^†^ | 0.003 |  | -0.021 | -0.008 | 1.077 |  |
|  | Physical activity level | 0.052 | 0.146 |  | -0.238 | 0.343 | 1.063 |  |
|  | ln fat mass_(DXA)_ (kg) | 0.034 | 0.070 |  | -0.105 | 0.172 | 1.28 |  |
|  |  |  |  |  |  |  |  |  |
| ***Dependent variable: 1RM (ln kg)*** | | | | | | | | |
| 1 | Constant | 3.703^*^ | 1.198 |  | 1.322 | 6.083 |  | 0.43 |
|  | Sex | 0.501^†^ | 0.083 |  | 0.336 | 0.666 | 1.129 |  |
|  | Age | -0.027^†^ | 0.006 |  | -0.039 | -0.015 | 1.181 |  |
|  | Physical activity level | -0.018 | 0.257 |  | -0.530 | 0.493 | 1.059 |  |
|  | ln body mass (kg) | 0.436 | 0.219 |  | 0.001 | 0.871 | 1.218 |  |
| 2 | Constant | 4.152^†^ | 0.693 |  | 2.774 | 5.530 |  | 0.48 |
|  | Sex | 0.305^*^ | 0.104 |  | 0.098 | 0.512 | 1.919 |  |
|  | Age | -0.025^†^ | 0.006 |  | -0.037 | -0.013 | 1.143 |  |
|  | Physical activity level | -0.137 | 0.249 |  | -0.631 | 0.357 | 1.068 |  |
|  | ln height (m) | 3.047^*^ | 0.897 |  | 1.266 | 4.829 | 1.891 |  |
| 3 | Constant | 4.546^*^ | 1.609 |  | 1.35 | 7.743 |  | 0.41 |
|  | Sex | 0.506^†^ | 0.092 |  | 0.323 | 0.688 | 1.334 |  |
|  | Age | -0.03^†^ | 0.006 |  | -0.042 | -0.018 | 1.128 |  |
|  | Physical activity level | -0.049 | 0.262 |  | -0.569 | 0.471 | 1.056 |  |
|  | ln forearm circumference (cm) | 0.384 | 0.441 |  | -0.492 | 1.260 | 1.349 |  |
| 4 | Constant | 1.472 | 1.788 |  | -2.081 | 5.025 |  | 0.45 |
|  | Sex | 0.511^†^ | 0.080 |  | 0.352 | 0.671 | 1.084 |  |
|  | Age | -0.026^†^ | 0.006 |  | -0.038 | -0.014 | 1.186 |  |
|  | Physical activity level | 0.03 | 0.255 |  | -0.477 | 0.538 | 1.071 |  |
|  | ln calf circumference (cm) | 1.104^*^ | 0.431 |  | 0.247 | 1.962 | 1.195 |  |
| 5 | Constant | 3.186 | 2.190 |  | -1.166 | 7.538 |  | 0.42 |
|  | Sex | 0.513^†^ | 0.085 |  | 0.345 | 0.681 | 1.143 |  |
|  | Age | -0.029^†^ | 0.006 |  | -0.041 | -0.017 | 1.110 |  |
|  | Physical activity level | -0.038 | 0.261 |  | -0.555 | 0.48 | 1.057 |  |
|  | ln chest circumference (cm) | 0.558 | 0.442 |  | -0.32 | 1.436 | 1.162 |  |
| 6 | Constant | 4.581^*^ | 1.553 |  | 1.496 | 7.667 |  | 0.41 |
|  | Sex | 0.524^†^ | 0.084 |  | 0.356 | 0.692 | 1.128 |  |
|  | Age | -0.031^†^ | 0.006 |  | -0.042 | -0.019 | 1.045 |  |
|  | Physical activity level | -0.046 | 0.262 |  | -0.566 | 0.474 | 1.056 |  |
|  | ln waist circumference (cm) | 0.277 | 0.315 |  | -0.347 | 0.902 | 1.088 |  |
| 7 | Constant | -3.215 | 3.276 |  | -9.725 | 3.295 |  | 0.46 |
|  | Sex | 0.360^*^ | 0.102 |  | 0.157 | 0.563 | 1.779 |  |
|  | Age | -0.028^†^ | 0.006 |  | -0.04 | -0.017 | 1.061 |  |
|  | Physical activity level | -0.094 | 0.252 |  | -0.595 | 0.408 | 1.061 |  |
|  | ln knee height (cm) | 2.284^*^ | 0.814 |  | 0.667 | 3.901 | 1.708 |  |
| 8 | Constant | -6.682 | 3.473 |  | -13.584 | 0.219 |  | 0.49 |
|  | Sex | 0.324^*^ | 0.097 |  | 0.130 | 0.517 | 1.705 |  |
|  | Age | -0.024^†^ | 0.006 |  | -0.036 | -0.012 | 1.161 |  |
|  | Physical activity level | 0.013 | 0.246 |  | -0.475 | 0.501 | 1.061 |  |
|  | ln half arm span (cm) | 2.728^†^ | 0.747 |  | 1.243 | 4.213 | 1.752 |  |
| 9 | Constant | 5.136^†^ | 0.651 |  | 3.842 | 6.430 |  | 0.43 |
|  | Sex | 0.647^†^ | 0.101 |  | 0.447 | 0.847 | 1.640 |  |
|  | Age | -0.03^†^ | 0.006 |  | -0.041 | -0.018 | 1.058 |  |
|  | Physical activity level | -0.022 | 0.259 |  | -0.536 | 0.492 | 1.059 |  |
|  | ln triceps skinfold (mm) | 0.179 | 0.103 |  | -0.025 | 0.383 | 1.637 |  |
| 10 | Constant | 5.585^†^ | 0.616 |  | 4.360 | 6.809 |  | 0.41 |
|  | Sex | 0.596^†^ | 0.105 |  | 0.387 | 0.805 | 1.752 |  |
|  | Age | -0.031^†^ | 0.006 |  | -0.043 | -0.019 | 1.041 |  |
|  | Physical activity level | -0.011 | 0.265 |  | -0.538 | 0.516 | 1.086 |  |
|  | ln biceps skinfold (mm) | 0.077 | 0.095 |  | -0.111 | 0.265 | 1.706 |  |
| 11 | Constant | 5.74^†^ | 0.650 |  | 4.448 | 7.033 |  | 0.41 |
|  | Sex | 0.563^†^ | 0.103 |  | 0.358 | 0.767 | 1.660 |  |
|  | Age | -0.031^†^ | 0.006 |  | -0.043 | -0.02 | 1.026 |  |
|  | Physical activity level | -0.031 | 0.267 |  | -0.561 | 0.499 | 1.091 |  |
|  | ln thigh skinfold (mm) | 0.032 | 0.095 |  | -0.158 | 0.222 | 1.595 |  |
| 12 | Constant | 5.806^†^ | 0.604 |  | 4.606 | 7.007 |  | 0.41 |
|  | Sex | 0.556^†^ | 0.105 |  | 0.347 | 0.765 | 1.737 |  |
|  | Age | -0.031^†^ | 0.006 |  | -0.043 | -0.02 | 1.041 |  |
|  | Physical activity level | -0.044 | 0.263 |  | -0.566 | 0.479 | 1.06 |  |
|  | ln medial calf skinfold (mm) | 0.018 | 0.082 |  | -0.146 | 0.181 | 1.711 |  |
| 13 | Constant | -0.722 | 2.76 |  | -6.207 | 4.763 |  | 0.45 |
|  | Sex | 0.417^†^ | 0.095 |  | 0.228 | 0.605 | 1.506 |  |
|  | Age | -0.024^†^ | 0.006 |  | -0.037 | -0.012 | 1.295 |  |
|  | Physical activity level | 0.023 | 0.256 |  | -0.486 | 0.532 | 1.070 |  |
|  | ln biacromial breadth (mm) | 1.668^*^ | 0.687 |  | 0.304 | 3.033 | 1.689 |  |
| 14 | Constant | 3.329 | 2.313 |  | -1.267 | 7.924 |  | 0.42 |
|  | Sex | 0.532^†^ | 0.082 |  | 0.369 | 0.695 | 1.072 |  |
|  | Age | -0.03^†^ | 0.006 |  | -0.042 | -0.018 | 1.101 |  |
|  | Physical activity level | -0.043 | 0.261 |  | -0.561 | 0.476 | 1.056 |  |
|  | ln bitrochanteric breadth (mm) | 0.688 | 0.610 |  | -0.523 | 1.900 | 1.091 |  |
| 15 | Constant | 3.52^*^ | 1.23 |  | 1.076 | 5.965 |  | 0.44 |
|  | Sex | 0.423^†^ | 0.098 |  | 0.228 | 0.619 | 1.593 |  |
|  | Age | -0.03^†^ | 0.006 |  | -0.041 | -0.018 | 1.043 |  |
|  | Physical activity level | -0.043 | 0.257 |  | -0.553 | 0.467 | 1.056 |  |
|  | ln bimalleolar breadth (mm) | 1.204^*^ | 0.576 |  | 0.059 | 2.349 | 1.538 |  |
| 16 | Constant | 5.702^†^ | 1.028 |  | 3.660 | 7.744 |  | 0.41 |
|  | Sex | 0.529^†^ | 0.107 |  | 0.316 | 0.741 | 1.792 |  |
|  | Age | -0.031^†^ | 0.006 |  | -0.043 | -0.02 | 1.037 |  |
|  | Physical activity level | -0.052 | 0.264 |  | -0.576 | 0.472 | 1.065 |  |
|  | ln elbow breadth (mm) | 0.096 | 0.489 |  | -0.874 | 1.067 | 1.704 |  |
| 17 | Constant | 5.159^†^ | 0.969 |  | 3.234 | 7.083 |  | 0.41 |
|  | Sex | 0.486^†^ | 0.104 |  | 0.279 | 0.693 | 1.715 |  |
|  | Age | -0.031^†^ | 0.006 |  | -0.043 | -0.02 | 1.018 |  |
|  | Physical activity level | -0.056 | 0.262 |  | -0.576 | 0.465 | 1.058 |  |
|  | ln wrist breadth (mm) | 0.440 | 0.508 |  | -0.568 | 1.449 | 1.638 |  |
| 18 | Constant | 3.383 | 2.075 |  | -0.74 | 7.506 |  | 0.42 |
|  | Sex | 0.469^†^ | 0.101 |  | 0.269 | 0.669 | 1.616 |  |
|  | Age | -0.029^†^ | 0.006 |  | -0.041 | -0.016 | 1.178 |  |
|  | Physical activity level | -0.024 | 0.261 |  | -0.543 | 0.494 | 1.061 |  |
|  | ln chest breadth (mm) | 0.684 | 0.552 |  | -0.413 | 1.781 | 1.679 |  |
| 19 | Constant | 3.212^*^ | 1.176 |  | 0.875 | 5.55 |  | 0.45 |
|  | Sex | 0.460^†^ | 0.086 |  | 0.289 | 0.631 | 1.243 |  |
|  | Age | -0.025^†^ | 0.006 |  | -0.038 | -0.013 | 1.207 |  |
|  | Physical activity level | -0.030 | 0.254 |  | -0.535 | 0.475 | 1.057 |  |
|  | ln body mass*height (kg*m) | 0.480^*^ | 0.193 |  | 0.097 | 0.863 | 1.339 |  |
| 20 | Constant | 4.952^†^ | 0.619 |  | 3.722 | 6.183 |  | 0.45 |
|  | Sex | 0.467^†^ | 0.086 |  | 0.297 | 0.637 | 1.222 |  |
|  | Age | -0.026^†^ | 0.006 |  | -0.038 | -0.013 | 1.203 |  |
|  | Physical activity level | -0.027 | 0.255 |  | -0.533 | 0.479 | 1.057 |  |
|  | ln SA (m²) | 0.925^*^ | 0.384 |  | 0.163 | 1.688 | 1.317 |  |
| 21 | Constant | 5.277^†^ | 1.258 |  | 2.777 | 7.777 |  | 0.41 |
|  | Sex | 0.523^†^ | 0.090 |  | 0.344 | 0.702 | 1.276 |  |
|  | Age | -0.031^†^ | 0.006 |  | -0.043 | -0.018 | 1.129 |  |
|  | Physical activity level | -0.058 | 0.263 |  | -0.581 | 0.465 | 1.062 |  |
|  | ln MAMC (cm) | 0.176 | 0.337 |  | -0.494 | 0.846 | 1.292 |  |
| 22 | Constant | 5.528^†^ | 0.772 |  | 3.993 | 7.062 |  | 0.41 |
|  | Sex | 0.529^†^ | 0.085 |  | 0.359 | 0.698 | 1.141 |  |
|  | Age | -0.030^†^ | 0.006 |  | -0.043 | -0.018 | 1.134 |  |
|  | Physical activity level | -0.059 | 0.263 |  | -0.582 | 0.463 | 1.063 |  |
|  | ln CAMA (cm²) | 0.082 | 0.137 |  | -0.19 | 0.353 | 1.18 |  |
| 23 | Constant | 1.932 | 1.625 |  | -1.297 | 5.161 |  | 0.45 |
|  | Sex | 0.234 | 0.145 |  | -0.054 | 0.522 | 3.528 |  |
|  | Age | -0.021^*^ | 0.007 |  | -0.035 | -0.008 | 1.529 |  |
|  | Physical activity level | 0.018 | 0.255 |  | -0.489 | 0.525 | 1.067 |  |
|  | ln FFM_(LEAN et al., 1996)_ (kg) | 0.879^*^ | 0.346 |  | 0.192 | 1.566 | 3.814 |  |
| 24 | Constant | 3.787^*^ | 1.603 |  | 0.603 | 6.972 |  | 0.42 |
|  | Sex | 0.427^†^ | 0.117 |  | 0.195 | 0.659 | 2.181 |  |
|  | Age | -0.028^†^ | 0.006 |  | -0.041 | -0.016 | 1.174 |  |
|  | Physical activity level | -0.056 | 0.260 |  | -0.573 | 0.460 | 1.057 |  |
|  | ln left arm LST (g) | 0.257 | 0.188 |  | -0.115 | 0.630 | 2.182 |  |
| 25 | Constant | 3.986^*^ | 1.698 |  | 0.613 | 7.359 |  | 0.42 |
|  | Sex | 0.444^†^ | 0.117 |  | 0.211 | 0.677 | 2.187 |  |
|  | Age | -0.028^†^ | 0.006 |  | -0.041 | -0.015 | 1.258 |  |
|  | Physical activity level | -0.032 | 0.261 |  | -0.550 | 0.487 | 1.059 |  |
|  | ln right arm LST (g) | 0.22 | 0.189 |  | -0.155 | 0.596 | 2.282 |  |
| 26 | Constant | -0.207 | 2.151 |  | -4.481 | 4.066 |  | 0.46 |
|  | Sex | 0.322^*^ | 0.109 |  | 0.105 | 0.539 | 2.049 |  |
|  | Age | -0.023^†^ | 0.006 |  | -0.036 | -0.011 | 1.275 |  |
|  | Physical activity level | -0.033 | 0.251 |  | -0.532 | 0.466 | 1.056 |  |
|  | ln left leg LST (g) | 0.639^*^ | 0.220 |  | 0.202 | 1.077 | 2.149 |  |
| 27 | Constant | 0.338 | 2.280 |  | -4.192 | 4.868 |  | 0.45 |
|  | Sex | 0.336^*^ | 0.115 |  | 0.108 | 0.564 | 2.211 |  |
|  | Age | -0.024^†^ | 0.006 |  | -0.037 | -0.012 | 1.288 |  |
|  | Physical activity level | 0.011 | 0.255 |  | -0.496 | 0.518 | 1.065 |  |
|  | ln right leg LST (g) | 0.577^*^ | 0.232 |  | 0.116 | 1.037 | 2.351 |  |
| 28 | Constant | 3.537 | 1.851 |  | -0.142 | 7.215 |  | 0.42 |
|  | Sex | 0.427^*^ | 0.120 |  | 0.189 | 0.665 | 2.287 |  |
|  | Age | -0.028^†^ | 0.006 |  | -0.041 | -0.015 | 1.237 |  |
|  | Physical activity level | -0.042 | 0.260 |  | -0.559 | 0.475 | 1.056 |  |
|  | ln arms LST (g) | 0.258 | 0.197 |  | -0.132 | 0.649 | 2.346 |  |
| 29 | Constant | -0.695 | 2.435 |  | -5.534 | 4.144 |  | 0.46 |
|  | Sex | 0.317^*^ | 0.114 |  | 0.092 | 0.543 | 2.194 |  |
|  | Age | -0.023^†^ | 0.006 |  | -0.036 | -0.011 | 1.298 |  |
|  | Physical activity level | -0.006 | 0.253 |  | -0.508 | 0.495 | 1.060 |  |
|  | ln legs LST (g) | 0.641^*^ | 0.233 |  | 0.178 | 1.103 | 2.324 |  |
| 30 | Constant | 3.848^†^ | 0.966 |  | 1.929 | 5.768 |  | 0.45 |
|  | Sex | 0.333^*^ | 0.117 |  | 0.100 | 0.566 | 2.299 |  |
|  | Age | -0.024^†^ | 0.006 |  | -0.037 | -0.012 | 1.302 |  |
|  | Physical activity level | -0.018 | 0.255 |  | -0.524 | 0.488 | 1.058 |  |
|  | ln ASM (kg) | 0.560^*^ | 0.231 |  | 0.102 | 1.018 | 2.422 |  |
| 31 | Constant | 5.305^†^ | 0.834 |  | 3.648 | 6.961 |  | 0.41 |
|  | Sex | 0.492^†^ | 0.101 |  | 0.292 | 0.692 | 1.601 |  |
|  | Age | -0.030^†^ | 0.006 |  | -0.042 | -0.017 | 1.179 |  |
|  | Physical activity level | -0.021 | 0.263 |  | -0.545 | 0.502 | 1.070 |  |
|  | ln ASM/height² (kg/m²) | 0.231 | 0.268 |  | -0.302 | 0.764 | 1.685 |  |
| 32 | Constant | 2.917^*^ | 1.443 |  | 0.05 | 5.785 |  | 0.45 |
|  | Sex | 0.415^†^ | 0.097 |  | 0.222 | 0.607 | 1.503 |  |
|  | Age | -0.027^†^ | 0.006 |  | -0.039 | -0.014 | 1.235 |  |
|  | Physical activity level | 0.012 | 0.259 |  | -0.502 | 0.526 | 1.074 |  |
|  | ln FFM_(BAUMGARTNER et al., 1991)_ (kg) | 0.671^*^ | 0.297 |  | 0.081 | 1.262 | 1.665 |  |
| 33 | Constant | 4.193^*^ | 1.251 |  | 1.709 | 6.678 |  | 0.42 |
|  | Sex | 0.431^†^ | 0.111 |  | 0.21 | 0.652 | 1.979 |  |
|  | Age | -0.028^†^ | 0.006 |  | -0.040 | -0.015 | 1.231 |  |
|  | Physical activity level | -0.034 | 0.260 |  | -0.550 | 0.482 | 1.057 |  |
|  | ln FFM_(DXA)_ (kg) | 0.384 | 0.261 |  | -0.136 | 0.903 | 2.054 |  |
|  |  |  |  |  |  |  |  |  |
| ***Dependent variable: PT^s^ (ln Nm)*** | | | | | | | | |
| 1 | Constant | 4.174^*^ | 1.230 |  | 1.731 | 6.618 |  | 0.40 |
|  | Sex | 0.520^†^ | 0.085 |  | 0.351 | 0.689 | 1.129 |  |
|  | Age | -0.025^†^ | 0.006 |  | -0.038 | -0.013 | 1.181 |  |
|  | Physical activity level | 0.259 | 0.264 |  | -0.267 | 0.784 | 1.059 |  |
|  | ln body mass (kg) | 0.367 | 0.225 |  | -0.08 | 0.814 | 1.218 |  |
| 2 | Constant | 4.153^†^ | 0.702 |  | 2.759 | 5.548 |  | 0.46 |
|  | Sex | 0.301^*^ | 0.105 |  | 0.091 | 0.510 | 1.919 |  |
|  | Age | -0.022^†^ | 0.006 |  | -0.034 | -0.010 | 1.143 |  |
|  | Physical activity level | 0.138 | 0.252 |  | -0.362 | 0.638 | 1.068 |  |
|  | ln height (m) | 3.268^*^ | 0.908 |  | 1.465 | 5.071 | 1.891 |  |
| 3 | Constant | 3.966^*^ | 1.631 |  | 0.725 | 7.206 |  | 0.40 |
|  | Sex | 0.499^†^ | 0.093 |  | 0.314 | 0.684 | 1.334 |  |
|  | Age | -0.026^†^ | 0.006 |  | -0.039 | -0.014 | 1.128 |  |
|  | Physical activity level | 0.232 | 0.265 |  | -0.295 | 0.759 | 1.056 |  |
|  | ln forearm circumference (cm) | 0.588 | 0.447 |  | -0.300 | 1.476 | 1.349 |  |
| 4 | Constant | 1.299 | 1.816 |  | -2.308 | 4.907 |  | 0.43 |
|  | Sex | 0.522^†^ | 0.082 |  | 0.360 | 0.683 | 1.084 |  |
|  | Age | -0.023^†^ | 0.006 |  | -0.035 | -0.010 | 1.186 |  |
|  | Physical activity level | 0.317 | 0.259 |  | -0.198 | 0.832 | 1.071 |  |
|  | ln calf circumference (cm) | 1.179^*^ | 0.438 |  | 0.309 | 2.05 | 1.195 |  |
| 5 | Constant | 2.509 | 2.220 |  | -1.901 | 6.920 |  | 0.40 |
|  | Sex | 0.517^†^ | 0.086 |  | 0.347 | 0.687 | 1.143 |  |
|  | Age | -0.026^†^ | 0.006 |  | -0.038 | -0.014 | 1.110 |  |
|  | Physical activity level | 0.247 | 0.264 |  | -0.278 | 0.771 | 1.057 |  |
|  | ln chest circumference (cm) | 0.724 | 0.448 |  | -0.166 | 1.614 | 1.162 |  |
| 6 | Constant | 4.120^*^ | 1.576 |  | 0.990 | 7.251 |  | 0.40 |
|  | Sex | 0.528^†^ | 0.086 |  | 0.358 | 0.698 | 1.128 |  |
|  | Age | -0.028^†^ | 0.006 |  | -0.04 | -0.016 | 1.045 |  |
|  | Physical activity level | 0.236 | 0.265 |  | -0.291 | 0.763 | 1.056 |  |
|  | ln waist circumference (cm) | 0.403 | 0.319 |  | -0.231 | 1.037 | 1.088 |  |
| 7 | Constant | -1.243 | 3.396 |  | -7.991 | 5.504 |  | 0.42 |
|  | Sex | 0.409^†^ | 0.106 |  | 0.199 | 0.620 | 1.779 |  |
|  | Age | -0.026^†^ | 0.006 |  | -0.038 | -0.015 | 1.061 |  |
|  | Physical activity level | 0.197 | 0.262 |  | -0.322 | 0.717 | 1.061 |  |
|  | ln knee height (cm) | 1.820^*^ | 0.843 |  | 0.144 | 3.496 | 1.708 |  |
| 8 | Constant | -1.433 | 3.711 |  | -8.807 | 5.940 |  | 0.41 |
|  | Sex | 0.425^†^ | 0.104 |  | 0.219 | 0.632 | 1.705 |  |
|  | Age | -0.025^†^ | 0.006 |  | -0.037 | -0.012 | 1.161 |  |
|  | Physical activity level | 0.270 | 0.262 |  | -0.252 | 0.791 | 1.061 |  |
|  | ln half arm span (cm) | 1.615^*^ | 0.799 |  | 0.029 | 3.202 | 1.752 |  |
| 9 | Constant | 5.377^†^ | 0.667 |  | 4.051 | 6.702 |  | 0.40 |
|  | Sex | 0.643^†^ | 0.103 |  | 0.438 | 0.848 | 1.640 |  |
|  | Age | -0.027^†^ | 0.006 |  | -0.039 | -0.015 | 1.058 |  |
|  | Physical activity level | 0.256 | 0.265 |  | -0.271 | 0.782 | 1.059 |  |
|  | ln triceps skinfold (mm) | 0.152 | 0.105 |  | -0.057 | 0.361 | 1.637 |  |
| 10 | Constant | 5.780^†^ | 0.629 |  | 4.530 | 7.030 |  | 0.39 |
|  | Sex | 0.596^†^ | 0.108 |  | 0.382 | 0.809 | 1.752 |  |
|  | Age | -0.028^†^ | 0.006 |  | -0.04 | -0.016 | 1.041 |  |
|  | Physical activity level | 0.262 | 0.271 |  | -0.277 | 0.800 | 1.086 |  |
|  | ln biceps skinfold (mm) | 0.059 | 0.097 |  | -0.133 | 0.251 | 1.706 |  |
| 11 | Constant | 6.422^†^ | 0.660 |  | 5.111 | 7.733 |  | 0.39 |
|  | Sex | 0.492^†^ | 0.104 |  | 0.285 | 0.700 | 1.660 |  |
|  | Age | -0.030^†^ | 0.006 |  | -0.041 | -0.018 | 1.026 |  |
|  | Physical activity level | 0.186 | 0.271 |  | -0.351 | 0.724 | 1.091 |  |
|  | ln thigh skinfold (mm) | -0.096 | 0.097 |  | -0.288 | 0.096 | 1.595 |  |
| 12 | Constant | 5.993^†^ | 0.616 |  | 4.77 | 7.217 |  | 0.39 |
|  | Sex | 0.557^†^ | 0.107 |  | 0.344 | 0.770 | 1.737 |  |
|  | Age | -0.029^†^ | 0.006 |  | -0.041 | -0.017 | 1.041 |  |
|  | Physical activity level | 0.235 | 0.268 |  | -0.298 | 0.768 | 1.060 |  |
|  | ln medial calf skinfold (mm) | 0.003 | 0.084 |  | -0.164 | 0.169 | 1.711 |  |
| 13 | Constant | -2.485 | 2.757 |  | -7.964 | 2.994 |  | 0.45 |
|  | Sex | 0.393^†^ | 0.095 |  | 0.205 | 0.582 | 1.506 |  |
|  | Age | -0.020^*^ | 0.006 |  | -0.032 | -0.007 | 1.295 |  |
|  | Physical activity level | 0.325 | 0.256 |  | -0.183 | 0.833 | 1.070 |  |
|  | ln biacromial breadth (mm) | 2.146^*^ | 0.686 |  | 0.783 | 3.509 | 1.689 |  |
| 14 | Constant | 4.151 | 2.365 |  | -0.548 | 8.851 |  | 0.39 |
|  | Sex | 0.547^†^ | 0.084 |  | 0.38 | 0.714 | 1.072 |  |
|  | Age | -0.028^†^ | 0.006 |  | -0.040 | -0.015 | 1.101 |  |
|  | Physical activity level | 0.238 | 0.267 |  | -0.293 | 0.768 | 1.056 |  |
|  | ln bitrochanteric breadth (mm) | 0.500 | 0.624 |  | -0.739 | 1.739 | 1.091 |  |
| 15 | Constant | 2.982^*^ | 1.236 |  | 0.527 | 5.438 |  | 0.43 |
|  | Sex | 0.402^†^ | 0.099 |  | 0.206 | 0.599 | 1.593 |  |
|  | Age | -0.026^†^ | 0.006 |  | -0.038 | -0.015 | 1.043 |  |
|  | Physical activity level | 0.239 | 0.258 |  | -0.273 | 0.751 | 1.056 |  |
|  | ln bimalleolar breadth (mm) | 1.543^*^ | 0.579 |  | 0.393 | 2.693 | 1.538 |  |
| 16 | Constant | 5.825^†^ | 1.047 |  | 3.744 | 7.906 |  | 0.39 |
|  | Sex | 0.541^†^ | 0.109 |  | 0.324 | 0.757 | 1.792 |  |
|  | Age | -0.029^†^ | 0.006 |  | -0.041 | -0.017 | 1.037 |  |
|  | Physical activity level | 0.230 | 0.269 |  | -0.304 | 0.764 | 1.065 |  |
|  | ln elbow breadth (mm) | 0.098 | 0.498 |  | -0.891 | 1.087 | 1.704 |  |
| 17 | Constant | 4.697^†^ | 0.978 |  | 2.754 | 6.640 |  | 0.40 |
|  | Sex | 0.453^†^ | 0.105 |  | 0.244 | 0.662 | 1.715 |  |
|  | Age | -0.029^†^ | 0.006 |  | -0.04 | -0.017 | 1.018 |  |
|  | Physical activity level | 0.219 | 0.264 |  | -0.306 | 0.744 | 1.058 |  |
|  | ln wrist breadth (mm) | 0.800 | 0.513 |  | -0.218 | 1.819 | 1.638 |  |
| 18 | Constant | 0.890 | 2.058 |  | -3.200 | 4.980 |  | 0.43 |
|  | Sex | 0.405^†^ | 0.100 |  | 0.206 | 0.603 | 1.616 |  |
|  | Age | -0.023^†^ | 0.006 |  | -0.035 | -0.011 | 1.178 |  |
|  | Physical activity level | 0.281 | 0.259 |  | -0.234 | 0.795 | 1.061 |  |
|  | ln chest breadth (mm) | 1.402^*^ | 0.548 |  | 0.314 | 2.490 | 1.679 |  |
| 19 | Constant | 3.591^*^ | 1.208 |  | 1.191 | 5.990 |  | 0.42 |
|  | Sex | 0.480^†^ | 0.088 |  | 0.304 | 0.656 | 1.243 |  |
|  | Age | -0.023^†^ | 0.006 |  | -0.036 | -0.011 | 1.207 |  |
|  | Physical activity level | 0.250 | 0.261 |  | -0.268 | 0.769 | 1.057 |  |
|  | ln body mass*height (kg*m) | 0.435^*^ | 0.198 |  | 0.041 | 0.828 | 1.339 |  |
| 20 | Constant | 5.174^†^ | 0.636 |  | 3.911 | 6.438 |  | 0.41 |
|  | Sex | 0.487^†^ | 0.088 |  | 0.313 | 0.662 | 1.222 |  |
|  | Age | -0.024^†^ | 0.006 |  | -0.036 | -0.011 | 1.203 |  |
|  | Physical activity level | 0.252 | 0.261 |  | -0.267 | 0.772 | 1.057 |  |
|  | ln SA (m²) | 0.830^*^ | 0.394 |  | 0.047 | 1.613 | 1.317 |  |
| 21 | Constant | 5.163^†^ | 1.281 |  | 2.618 | 7.708 |  | 0.39 |
|  | Sex | 0.528^†^ | 0.092 |  | 0.345 | 0.710 | 1.276 |  |
|  | Age | -0.028^†^ | 0.006 |  | -0.040 | -0.015 | 1.129 |  |
|  | Physical activity level | 0.219 | 0.268 |  | -0.313 | 0.752 | 1.062 |  |
|  | ln MAMC (cm) | 0.246 | 0.343 |  | -0.436 | 0.928 | 1.292 |  |
| 22 | Constant | 5.547^†^ | 0.786 |  | 3.985 | 7.108 |  | 0.39 |
|  | Sex | 0.537^†^ | 0.087 |  | 0.365 | 0.709 | 1.141 |  |
|  | Age | -0.027^†^ | 0.006 |  | -0.04 | -0.015 | 1.134 |  |
|  | Physical activity level | 0.218 | 0.268 |  | -0.313 | 0.750 | 1.063 |  |
|  | ln CAMA (cm²) | 0.107 | 0.139 |  | -0.17 | 0.383 | 1.180 |  |
| 23 | Constant | 3.606^*^ | 1.695 |  | 0.239 | 6.973 |  | 0.40 |
|  | Sex | 0.367^*^ | 0.151 |  | 0.067 | 0.667 | 3.528 |  |
|  | Age | -0.023^*^ | 0.007 |  | -0.037 | -0.008 | 1.529 |  |
|  | Physical activity level | 0.274 | 0.266 |  | -0.254 | 0.802 | 1.067 |  |
|  | ln FFM_(LEAN et al., 1996)_ (kg) | 0.534 | 0.36 |  | -0.182 | 1.250 | 3.814 |  |
| 24 | Constant | 3.378^*^ | 1.624 |  | 0.151 | 6.606 |  | 0.41 |
|  | Sex | 0.410^*^ | 0.118 |  | 0.175 | 0.645 | 2.181 |  |
|  | Age | -0.025^†^ | 0.006 |  | -0.038 | -0.013 | 1.174 |  |
|  | Physical activity level | 0.223 | 0.264 |  | -0.301 | 0.746 | 1.057 |  |
|  | ln left arm LST (g) | 0.323 | 0.19 |  | -0.054 | 0.701 | 2.182 |  |
| 25 | Constant | 5.207^*^ | 1.741 |  | 1.747 | 8.666 |  | 0.39 |
|  | Sex | 0.513^†^ | 0.120 |  | 0.274 | 0.752 | 2.187 |  |
|  | Age | -0.028^†^ | 0.007 |  | -0.041 | -0.014 | 1.258 |  |
|  | Physical activity level | 0.241 | 0.268 |  | -0.291 | 0.773 | 1.059 |  |
|  | ln right arm LST (g) | 0.093 | 0.194 |  | -0.292 | 0.478 | 2.282 |  |
| 26 | Constant | 1.938 | 2.25 |  | -2.533 | 6.409 |  | 0.41 |
|  | Sex | 0.407^*^ | 0.114 |  | 0.18 | 0.634 | 2.049 |  |
|  | Age | -0.024^*^ | 0.007 |  | -0.037 | -0.010 | 1.275 |  |
|  | Physical activity level | 0.244 | 0.263 |  | -0.278 | 0.766 | 1.056 |  |
|  | ln left leg LST (g) | 0.427 | 0.23 |  | -0.031 | 0.885 | 2.149 |  |
| 27 | Constant | 1.383 | 2.35 |  | -3.286 | 6.052 |  | 0.41 |
|  | Sex | 0.383^*^ | 0.118 |  | 0.147 | 0.618 | 2.211 |  |
|  | Age | -0.023^*^ | 0.007 |  | -0.036 | -0.010 | 1.288 |  |
|  | Physical activity level | 0.283 | 0.263 |  | -0.240 | 0.805 | 1.065 |  |
|  | ln right leg LST (g) | 0.481^*^ | 0.239 |  | 0.006 | 0.956 | 2.351 |  |
| 28 | Constant | 4.074^*^ | 1.893 |  | 0.313 | 7.835 |  | 0.39 |
|  | Sex | 0.460^†^ | 0.122 |  | 0.217 | 0.703 | 2.287 |  |
|  | Age | -0.026^†^ | 0.007 |  | -0.039 | -0.013 | 1.237 |  |
|  | Physical activity level | 0.239 | 0.266 |  | -0.29 | 0.768 | 1.056 |  |
|  | ln arms LST (g) | 0.213 | 0.201 |  | -0.187 | 0.612 | 2.346 |  |
| 29 | Constant | 1.137 | 2.531 |  | -3.892 | 6.167 |  | 0.41 |
|  | Sex | 0.388^*^ | 0.118 |  | 0.154 | 0.623 | 2.194 |  |
|  | Age | -0.023^*^ | 0.007 |  | -0.036 | -0.010 | 1.298 |  |
|  | Physical activity level | 0.264 | 0.263 |  | -0.257 | 0.786 | 1.060 |  |
|  | ln legs LST (g) | 0.474 | 0.242 |  | -0.006 | 0.955 | 2.324 |  |
| 30 | Constant | 4.476^†^ | 0.999 |  | 2.491 | 6.462 |  | 0.41 |
|  | Sex | 0.397^*^ | 0.121 |  | 0.156 | 0.638 | 2.299 |  |
|  | Age | -0.023^*^ | 0.007 |  | -0.037 | -0.010 | 1.302 |  |
|  | Physical activity level | 0.257 | 0.263 |  | -0.267 | 0.780 | 1.058 |  |
|  | ln ASM (kg) | 0.422 | 0.238 |  | -0.052 | 0.896 | 2.422 |  |
| 31 | Constant | 5.959^†^ | 0.853 |  | 4.263 | 7.654 |  | 0.39 |
|  | Sex | 0.551^†^ | 0.103 |  | 0.346 | 0.755 | 1.601 |  |
|  | Age | -0.029^†^ | 0.006 |  | -0.042 | -0.016 | 1.179 |  |
|  | Physical activity level | 0.236 | 0.269 |  | -0.299 | 0.772 | 1.070 |  |
|  | ln ASM/height² (kg/m²) | 0.019 | 0.274 |  | -0.527 | 0.564 | 1.685 |  |
| 32 | Constant | 3.261^*^ | 1.486 |  | 0.306 | 6.215 |  | 0.41 |
|  | Sex | 0.443^†^ | 0.100 |  | 0.245 | 0.642 | 1.503 |  |
|  | Age | -0.024^*^ | 0.007 |  | -0.037 | -0.011 | 1.235 |  |
|  | Physical activity level | 0.294 | 0.266 |  | -0.236 | 0.823 | 1.074 |  |
|  | ln FFM_(BAUMGARTNER et al., 1991)_ (kg) | 0.612^*^ | 0.306 |  | 0.004 | 1.220 | 1.665 |  |
| 33 | Constant | 4.617^*^ | 1.280 |  | 2.074 | 7.160 |  | 0.40 |
|  | Sex | 0.463^†^ | 0.114 |  | 0.237 | 0.689 | 1.979 |  |
|  | Age | -0.026^†^ | 0.007 |  | -0.039 | -0.013 | 1.231 |  |
|  | Physical activity level | 0.245 | 0.266 |  | -0.283 | 0.773 | 1.057 |  |
|  | ln FFM_(DXA)_ (kg) | 0.316 | 0.268 |  | -0.216 | 0.848 | 2.054 |  |

^*^p<0,05 and ^†^p<0,001 (statistically significant β)

*Note*: sex=0 for women and 1 for men; age in years; physical activity level=0 for inactive and 1 for active; ln=natural logarithm; β=coefficient of regression; CI=confidence interval; LL=lower limit; UL=upper limit; VIF=variance inflation factor; R²=coefficient of determination; BMI=body mass index; SA=surface area of human body; MAMC=mid-arm muscle circumference; CAMA=corrected arm muscle area; AFA=arm fat area; FFM=Fat-free mass; LST=lean soft tissue; ASM=appendicular skeletal muscle mass; DXA=Dual-energy X-ray absorptiometry.

SUPPLEMENT D – Cut-off points to identify muscle weakness in older adults of the handgrip strength (HGS), one maximum repetition measurement for knee extensors (1RM) and isokinetic knee extension peak torque at 60º/s (PT) (non-normalized, ratio standard/muscle quality and allometric scaling), and the correlation of muscle strength with body size.

| **Variable** | **Unit** | **Men** | | | | |  | **Women** | | | | |  | **Correlation (r)**  **with body size** | | |
| --- | --- | --- | --- | --- | --- | --- | --- | --- | --- | --- | --- | --- | --- | --- | --- | --- |
|  |  | **AUC** | **95% CI** | **Cut-off point (≤)** | **Sens (%)** | **Spe (%)** |  | **AUC** | **95% CI** | **Cut-off point (≤)** | **Sens (%)** | **Spe (%)** |  | **Body mass** | **Height** | **Variable for normalization** |
|  |  |  |  |  |  |  |  |  |  |  |  |  |  |  |  |  |
| ***HGS (kg)*** | |  |  |  |  |  |  |  |  |  |  |  |  |  |  |  |
| Non-normalized |  | 0.74^*^ | 0.548 to 0.886 | 36 | 86 | 59 |  | 0.61 | 0.477 to 0.725 | 22 | 52 | 70 |  | 0.17 | 0.39^§^ | - |
| /body mass | (kg) | 0.66 | 0.464 to 0.826 | 0.54 | 86 | 45 |  | 0.76^†^ | 0.634 to 0.854 | 0.38 | 88 | 63 |  | -0.57^§^ | -0.03 | -0.57^§^ |
| /body mass^0.22^ |  | 0.73^*^ | 0.538 to 0.880 | 14.4 | 86 | 59 |  | 0.65^*^ | 0.523 to 0.765 | 9.5 | 68 | 65 |  | -0.02 | 0.29^‡^ | -0.02 |
| /body mass^0.40 (FOLEY et al., 1999)^ |  | 0.70 | 0.504 to 0.856 | 6.7 | 86 | 50 |  | 0.68^*^ | 0.556 to 0.793 | 4.41 | 72 | 68 |  | -0.18 | 0.22^‡^ | -0.18 |
| /body mass^0.67 (JARIC, 2003)^ |  | 0.70 | 0.504 to 0.856 | 2.15 | 86 | 50 |  | 0.73^*^ | 0.607 to 0.833 | 1.46 | 80 | 65 |  | -0.39^§^ | 0.10 | -0.39^§^ |
| /body mass^0.63 (PUA, 2006)^ |  | 0.70 | 0.504 to 0.856 | 2.55 | 86 | 50 |  | 0.73^*^ | 0.600 to 0.829 | 1.77 | 84 | 60 |  | -0.36^§^ | 0.11 | -0.36^§^ |
| /body mass^0.31 (MARANHÃO NETO et al., 2017)^ |  | 0.71^*^ | 0.517 to 0.865 | 10.9 | 100 | 41 |  | 0.67^*^ | 0.540 to 0.780 | 6.5 | 68 | 65 |  | -0.10 | 0.26^‡^ | -0.10 |
| /height | (m) | 0.70 | 0.504 to 0.856 | 23.5 | 100 | 45 |  | 0.60 | 0.474 to 0.722 | 15.8 | 72 | 50 |  | 0.09 | 0.17 | 0.17 |
| /height^1.87^ |  | 0.65 | 0.451 to 0.816 | 14.8 | 86 | 50 |  | 0.60 | 0.466 to 0.715 | 10.2 | 60 | 63 |  | 0.01 | -0.03 | -0.03 |
| /height^1.84 (MARANHÃO NETO et al., 2017)^ |  | 0.65 | 0.451 to 0.816 | 15.1 | 86 | 50 |  | 0.60 | 0.467 to 0.716 | 10.4 | 60 | 63 |  | 0.01 | -0.02 | -0.02 |
| /forearm circumference | (cm) | 0.74^*^ | 0.548 to 0.886 | 1.33 | 86 | 59 |  | 0.70^*^ | 0.570 to 0.805 | 1.04 | 84 | 58 |  | -0.17 | 0.27^‡^ | -0.19 |
| /forearm circumference^0.50^ |  | 0.75^*^ | 0.552 to 0.889 | 6.91 | 86 | 68 |  | 0.67^*^ | 0.537 to 0.777 | 4.84 | 68 | 65 |  | 0.01 | 0.34^‡^ | 0.02 |
| /calf circumference |  | 0.73^*^ | 0.531 to 0.875 | 0.92 | 57 | 82 |  | 0.67^*^ | 0.544 to 0.783 | 0.69 | 72 | 63 |  | -0.14 | 0.25^‡^ | -0.21 |
| /calf circumference^0.54^ |  | 0.73^*^ | 0.531 to 0.875 | 5.35 | 86 | 59 |  | 0.64^*^ | 0.515 to 0.758 | 3.54 | 64 | 60 |  | 0.01 | 0.32^‡^ | 0.01 |
| /chest circumference |  | 0.69 | 0.490 to 0.846 | 0.37 | 86 | 55 |  | 0.68^*^ | 0.551 to 0.789 | 0.28 | 84 | 58 |  | -0.20 | 0.24^‡^ | -0.39^§^ |
| /chest circumference^0.08^ |  | 0.75^*^ | 0.552 to 0.889 | 24.6 | 86 | 68 |  | 0.62 | 0.487 to 0.734 | 16.4 | 60 | 68 |  | 0.14 | 0.37^§^ | -0.02 |
| /knee height |  | 0.71 | 0.510 to 0.861 | 0.72 | 100 | 45 |  | 0.61 | 0.478 to 0.726 | 0.44 | 44 | 75 |  | 0.05 | 0.19 | 0.03 |
| /knee height^1.32^ |  | 0.70 | 0.504 to 0.856 | 0.21 | 100 | 50 |  | 0.61 | 0.482 to 0.729 | 0.13 | 44 | 78 |  | 0.01 | 0.13 | -0.05 |
| /half arm span |  | 0.69 | 0.490 to 0.846 | 0.47 | 100 | 36 |  | 0.59 | 0.464 to 0.713 | 0.28 | 48 | 75 |  | 0.11 | 0.21 | 0.19 |
| /half arm span^1.81^ |  | 0.65 | 0.451 to 0.816 | 0.012 | 100 | 32 |  | 0.58 | 0.453 to 0.703 | 0.0086 | 72 | 45 |  | 0.04 | 0.04 | -0.03 |
| /triceps skinfold | (mm) | 0.71^*^ | 0.514 to 0.863 | 2.25 | 86 | 59 |  | 0.65 | 0.519 to 0.762 | 0.93 | 64 | 68 |  | -0.30^‡^ | 0.15 | -0.55^§^ |
| /biceps skinfold |  | 0.63 | 0.428 to 0.798 | 6.67 | 100 | 36 |  | 0.73^*^ | 0.605 to 0.832 | 1.29 | 56 | 85 |  | -0.35^§^ | 0.16 | -0.53^§^ |
| /biceps skinfold^-0.02^ |  | 0.75^*^ | 0.552 to 0.889 | 41.6 | 100 | 45 |  | 0.59 | 0.459 to 0.709 | 23.2 | 48 | 78 |  | 0.21^‡^ | 0.38^§^ | -0.05 |
| /midaxillary skinfold |  | 0.62 | 0.419 to 0.790 | 0.86 | 29 | 100 |  | 0.52 | 0.395 to 0.648 | 1.23 | 80 | 33 |  | -0.34^‡^ | 0.12 | -0.65^§^ |
| /midaxillary skinfold^-0.01^ |  | 0.75^*^ | 0.552 to 0.889 | 40.7 | 100 | 45 |  | 0.61 | 0.479 to 0.727 | 22.5 | 48 | 78 |  | 0.19 | 0.38^§^ | -0.07 |
| /pectoral skinfold |  | 0.58 | 0.388 to 0.763 | 1.13 | 29 | 100 |  | 0.74^†^ | 0.618 to 0.843 | 1.64 | 76 | 73 |  | -0.31^‡^ | 0.21^‡^ | -0.70^§^ |
| /suprailiac skinfold |  | 0.61 | 0.413 to 0.785 | 1.88 | 71 | 55 |  | 0.62 | 0.488 to 0.735 | 0.77 | 64 | 73 |  | -0.34^‡^ | 0.12 | -0.60^§^ |
| /suprailiac skinfold^0.01^ |  | 0.74^*^ | 0.545 to 0.884 | 35.3 | 86 | 68 |  | 0.62 | 0.486 to 0.733 | 23.4 | 64 | 68 |  | 0.17 | 0.38^§^ | -0.07 |
| /abdominal skinfold |  | 0.66 | 0.464 to 0.826 | 1.13 | 57 | 73 |  | 0.69^*^ | 0.564 to 0.800 | 0.62 | 56 | 85 |  | -0.29^‡^ | 0.12 | -0.58^§^ |
| /thigh skinfold |  | 0.78^*^ | 0.584 to 0.909 | 1.82 | 71 | 77 |  | 0.81^†^ | 0.694 to 0.897 | 0.67 | 72 | 80 |  | -0.16 | 0.25^‡^ | -0.46^§^ |
| /medial calf skinfold |  | 0.80^†^ | 0.609 to 0.924 | 2.91 | 100 | 64 |  | 0.76^†^ | 0.632 to 0.853 | 0.93 | 64 | 78 |  | -0.15 | 0.16 | -0.42^§^ |
| /medial calf skinfold^-0.03^ |  | 0.73^*^ | 0.538 to 0.880 | 39.1 | 86 | 59 |  | 0.59 | 0.457 to 0.706 | 24.5 | 52 | 70 |  | 0.21 | 0.38^§^ | 0.04 |
| /biacromial breadth | (mm) | 0.68 | 0.484 to 0.841 | 1.02 | 100 | 36 |  | 0.62 | 0.493 to 0.739 | 0.67 | 76 | 50 |  | 0.01 | 0.26^‡^ | 0.01 |
| /biacromial breadth^1.06^ |  | 0.67 | 0.470 to 0.831 | 0.82 | 100 | 36 |  | 0.63 | 0.496 to 0.742 | 0.54 | 76 | 50 |  | 0.01 | 0.25^‡^ | -0.01 |
| /bitrochanteric breadth |  | 0.71 | 0.510 to 0.861 | 1.06 | 86 | 59 |  | 0.67^*^ | 0.538 to 0.778 | 0.7 | 68 | 60 |  | -0.02 | 0.29^‡^ | -0.05 |
| /bitrochanteric breadth^0.97^ |  | 0.71 | 0.510 to 0.861 | 1.18 | 86 | 59 |  | 0.67^*^ | 0.537 to 0.777 | 0.78 | 68 | 60 |  | -0.02 | 0.29^‡^ | -0.04 |
| /bimalleolar breadth |  | 0.65 | 0.454 to 0.819 | 6.25 | 100 | 32 |  | 0.66^*^ | 0.529 to 0.770 | 3.85 | 68 | 58 |  | 0.04 | 0.21^‡^ | -0.10 |
| /bimalleolar breadth^0.91^ |  | 0.68 | 0.480 to 0.839 | 6.78 | 86 | 50 |  | 0.65^*^ | 0.525 to 0.767 | 4.51 | 68 | 58 |  | 0.05 | 0.23^‡^ | -0.07 |
| /elbow breadth |  | 0.70 | 0.497 to 0.851 | 4.86 | 71 | 68 |  | 0.68^*^ | 0.550 to 0.788 | 4.18 | 76 | 58 |  | -0.04 | 0.31^‡^ | -0.26^‡^ |
| /elbow breadth^0.37^ |  | 0.73^*^ | 0.531 to 0.875 | 20.4 | 100 | 41 |  | 0.64 | 0.506 to 0.751 | 12.1 | 64 | 65 |  | 0.10 | 0.36^§^ | -0.03 |
| /wrist breadth |  | 0.80^*^ | 0.605 to 0.922 | 7.02 | 100 | 50 |  | 0.61 | 0.478 to 0.726 | 4.71 | 64 | 63 |  | 0.01 | 0.31^‡^ | -0.19 |
| /wrist breadth^0.58^ |  | 0.76^*^ | 0.562 to 0.896 | 14.7 | 100 | 45 |  | 0.60 | 0.474 to 0.722 | 9.5 | 64 | 60 |  | 0.08 | 0.35^§^ | -0.04 |
| /chest breadth |  | 0.67 | 0.470 to 0.831 | 1.32 | 100 | 45 |  | 0.60 | 0.466 to 0.716 | 0.88 | 64 | 63 |  | -0.03 | 0.28^‡^ | -0.20 |
| /chest breadth^0.34^ |  | 0.71^*^ | 0.517 to 0.865 | 11.5 | 86 | 59 |  | 0.61 | 0.476 to 0.725 | 7.53 | 56 | 70 |  | 0.04 | 0.31^‡^ | -0.08 |
| /body mass*height | (kg*m) | 0.60 | 0.400 to 0.774 | 0.17 | 29 | 100 |  | 0.74^†^ | 0.616 to 0.841 | 0.23 | 76 | 68 |  | -0.63^§^ | -0.19 | -0.60^§^ |
| /(body mass*height)^0.26^ |  | 0.70 | 0.497 to 0.851 | 12 | 100 | 36 |  | 0.66^*^ | 0.533 to 0.774 | 7.63 | 76 | 55 |  | -0.08 | 0.23^‡^ | -0.03 |
| /SA | (m²) | 0.69 | 0.490 to 0.846 | 20.8 | 86 | 50 |  | 0.70^*^ | 0.574 to 0.807 | 14.1 | 80 | 63 |  | -0.32^‡^ | 0.08 | -0.28^‡^ |
| /SA^0.49^ |  | 0.71 | 0.510 to 0.861 | 30.2 | 100 | 36 |  | 0.66^*^ | 0.529 to 0.770 | 18.3 | 68 | 63 |  | -0.07 | 0.24^‡^ | -0.02 |
| /MAMC | (cm) | 0.77^*^ | 0.573 to 0.902 | 1.39 | 71 | 77 |  | 0.70^*^ | 0.568 to 0.803 | 1.02 | 64 | 78 |  | -0.21^‡^ | 0.23^‡^ | -0.37^§^ |
| /MAMC^0.21^ |  | 0.75^*^ | 0.552 to 0.889 | 18.3 | 86 | 68 |  | 0.63 | 0.505 to 0.750 | 12.3 | 60 | 70 |  | 0.09 | 0.36^§^ | 0.08 |
| /CAMA | (cm²) | 0.70 | 0.504 to 0.856 | 0.83 | 71 | 73 |  | 0.71^*^ | 0.588 to 0.819 | 0.66 | 60 | 80 |  | -0.49^§^ | 0.01 | -0.74^§^ |
| /CAMA^0.08^ |  | 0.75^*^ | 0.552 to 0.889 | 26.3 | 86 | 68 |  | 0.63 | 0.504 to 0.749 | 17.6 | 60 | 70 |  | 0.10 | 0.36^§^ | 0.10 |
| /AFA |  | 0.71 | 0.517 to 0.865 | 2.17 | 86 | 55 |  | 0.70^*^ | 0.572 to 0.806 | 0.95 | 60 | 78 |  | -0.42^§^ | 0.08 | -0.59^§^ |
| /AFA^0.01^ |  | 0.74^*^ | 0.545 to 0.884 | 34.9 | 86 | 68 |  | 0.63 | 0.497 to 0.743 | 23.2 | 60 | 70 |  | 0.16 | 0.38^§^ | -0.01 |
| /FFM_(LEAN et al., 1996)_ | (kg) | 0.68 | 0.477 to 0.836 | 0.75 | 86 | 45 |  | 0.70^*^ | 0.577 to 0.810 | 0.68 | 84 | 63 |  | -0.32^‡^ | 0.06 | -0.27^‡^ |
| /FFM^0.53^_(LEAN et al., 1996)_ |  | 0.71 | 0.510 to 0.861 | 4.96 | 100 | 36 |  | 0.67^*^ | 0.544 to 0.783 | 3.63 | 76 | 58 |  | -0.09 | 0.22^‡^ | 0.01 |
| /fat mass_(LEAN et al., 1996)_ |  | 0.64 | 0.438 to 0.806 | 2.69 | 100 | 32 |  | 0.76^†^ | 0.638 to 0.857 | 0.8 | 76 | 78 |  | -0.62^§^ | -0.08 | -0.63^§^ |
| /fat mass^0.05^_(LEAN et al., 1996)_ |  | 0.75^*^ | 0.552 to 0.889 | 31.3 | 86 | 68 |  | 0.62 | 0.494 to 0.740 | 20.3 | 64 | 65 |  | 0.10 | 0.35^§^ | 0.02 |
| /left arm LST | (g) | 0.53 | 0.333 to 0.713 | 0.01 | 29 | 100 |  | 0.69^*^ | 0.558 to 0.795 | 0.016 | 72 | 73 |  | -0.42^§^ | 0.01 | -0.58^§^ |
| /left arm LST^0.29^ |  | 0.70 | 0.497 to 0.851 | 4.31 | 100 | 36 |  | 0.66^*^ | 0.528 to 0.769 | 2.93 | 72 | 63 |  | -0.02 | 0.29^‡^ | 0.05 |
| /right arm LST |  | 0.56 | 0.363 to 0.741 | 0.012 | 57 | 73 |  | 0.70^*^ | 0.576 to 0.809 | 0.013 | 68 | 80 |  | -0.4^§^ | 0.06 | -0.57^§^ |
| /right arm LST^0.26^ |  | 0.70 | 0.497 to 0.851 | 4.21 | 71 | 68 |  | 0.66^*^ | 0.531 to 0.772 | 3.3 | 68 | 65 |  | 0.01 | 0.32^‡^ | 0.08 |
| /left leg LST |  | 0.57 | 0.375 to 0.752 | 0.0027 | 29 | 100 |  | 0.65^*^ | 0.522 to 0.764 | 0.0041 | 60 | 75 |  | -0.41^§^ | -0.09 | -0.50^§^ |
| /left leg LST^0.30^ |  | 0.70 | 0.504 to 0.856 | 2.88 | 100 | 45 |  | 0.63 | 0.498 to 0.744 | 1.9 | 72 | 60 |  | -0.02 | 0.24^‡^ | 0.01 |
| /right leg LST |  | 0.53 | 0.333 to 0.713 | 0.0026 | 29 | 100 |  | 0.66^*^ | 0.530 to 0.771 | 0.0044 | 76 | 58 |  | -0.37^§^ | -0.01 | -0.47^§^ |
| /right leg LST^0.30^ |  | 0.68 | 0.477 to 0.836 | 2.65 | 100 | 36 |  | 0.63 | 0.504 to 0.749 | 1.72 | 64 | 63 |  | 0.01 | 0.27^‡^ | 0.05 |
| /arms LST |  | 0.54 | 0.345 to 0.725 | 0.0045 | 29 | 100 |  | 0.70^*^ | 0.573 to 0.807 | 0.0068 | 68 | 80 |  | -0.42^§^ | 0.04 | -0.55^§^ |
| /arms LST^0.30^ |  | 0.68 | 0.484 to 0.841 | 2.68 | 71 | 68 |  | 0.66^*^ | 0.528 to 0.769 | 2.09 | 64 | 68 |  | -0.03 | 0.30^‡^ | 0.07 |
| /legs LST |  | 0.55 | 0.357 to 0.736 | 0.0013 | 29 | 100 |  | 0.65^*^ | 0.518 to 0.761 | 0.002 | 60 | 75 |  | -0.39^§^ | -0.05 | -0.46^§^ |
| /legs LST^0.32^ |  | 0.68 | 0.477 to 0.836 | 1.91 | 100 | 36 |  | 0.64 | 0.506 to 0.751 | 1.25 | 68 | 63 |  | -0.02 | 0.25^‡^ | 0.03 |
| /ASM | (kg) | 0.54 | 0.345 to 0.725 | 1.03 | 29 | 100 |  | 0.66^*^ | 0.535 to 0.776 | 1.59 | 64 | 75 |  | -0.41^§^ | -0.03 | -0.46^§^ |
| /ASM^0.33^ |  | 0.66 | 0.464 to 0.826 | 14.9 | 100 | 32 |  | 0.65^*^ | 0.517 to 0.760 | 9.87 | 72 | 63 |  | -0.04 | 0.25^‡^ | 0.04 |
| /ASM/height² | (kg/m²) | 0.67 | 0.467 to 0.829 | 4.74 | 71 | 68 |  | 0.67^*^ | 0.538 to 0.778 | 4.17 | 76 | 65 |  | -0.23^‡^ | 0.34^‡^ | -0.50^§^ |
| /ASM/height²^(0.13)^ |  | 0.73^*^ | 0.531 to 0.875 | 31.3 | 100 | 41 |  | 0.63 | 0.499 to 0.745 | 18.9 | 64 | 65 |  | 0.12 | 0.38^§^ | 0.06 |
| /FFM_(BAUMGARTNER et al., 1991)_ | (kg) | 0.64 | 0.434 to 0.808 | 0.71 | 83 | 50 |  | 0.74^†^ | 0.619 to 0.845 | 0.56 | 88 | 59 |  | -0.4^§^ | 0.10 | -0.39^§^ |
| /FFM^0.41^_(BAUMGARTNER et al., 1991)_ |  | 0.69 | 0.488 to 0.849 | 7.11 | 83 | 59 |  | 0.68^*^ | 0.546 to 0.787 | 5.29 | 84 | 51 |  | -0.07 | 0.28^‡^ | -0.02 |
| /FFM_(DXA)_ |  | 0.60 | 0.406 to 0.779 | 0.41 | 29 | 100 |  | 0.73^*^ | 0.600 to 0.829 | 0.65 | 84 | 63 |  | -0.43^§^ | -0.04 | -0.44^§^ |
| /FFM^0.36^_(DXA)_ |  | 0.70 | 0.504 to 0.856 | 10.1 | 100 | 36 |  | 0.65^*^ | 0.526 to 0.768 | 6.71 | 72 | 60 |  | -0.06 | 0.24^‡^ | 0.02 |
| /fat mass_(DXA)_ |  | 0.71 | 0.517 to 0.865 | 1.01 | 43 | 95 |  | 0.77^†^ | 0.643 to 0.861 | 0.9 | 80 | 68 |  | -0.53^§^ | 0.03 | -0.66^§^ |
| /fat mass^0.03^_(DXA)_ |  | 0.74^*^ | 0.545 to 0.884 | 32.3 | 86 | 68 |  | 0.62 | 0.490 to 0.737 | 21.3 | 60 | 65 |  | 0.13 | 0.37^§^ | -0.04 |
|  |  |  |  |  |  |  |  |  |  |  |  |  |  |  |  |  |
| ***1RM (kg)*** | |  |  |  |  |  |  |  |  |  |  |  |  |  |  |  |
| Non-normalized |  | 0.75^*^ | 0.555 to 0.891 | 56.1 | 86 | 77 |  | 0.67^*^ | 0.545 to 0.784 | 38.1 | 72 | 60 |  | 0.18 | 0.33^‡^ | - |
| /body mass | (kg) | 0.77^*^ | 0.580 to 0.907 | 0.85 | 86 | 68 |  | 0.76^†^ | 0.634 to 0.854 | 0.54 | 68 | 78 |  | -0.28^‡^ | 0.13 | -0.28^‡^ |
| /body mass^0.44^ |  | 0.75^*^ | 0.559 to 0.893 | 9.04 | 86 | 77 |  | 0.71^*^ | 0.586 to 0.818 | 6.03 | 76 | 63 |  | -0.03 | 0.24^‡^ | -0.03 |
| /body mass^0.67 (JARIC, 2003)^ |  | 0.78^*^ | 0.587 to 0.911 | 3.4 | 86 | 77 |  | 0.73^†^ | 0.604 to 0.832 | 2.28 | 76 | 65 |  | -0.13 | 0.20 | -0.13 |
| /body mass^0.96 (ABDALLA et al., 2020)^ |  | 0.77^*^ | 0.573 to 0.902 | 1.00 | 86 | 68 |  | 0.75^†^ | 0.629 to 0.851 | 0.45 | 44 | 100 |  | -0.26^‡^ | 0.13 | -0.26^‡^ |
| /body mass^0.69 (ABDALLA et al., 2020)^ |  | 0.78^*^ | 0.587 to 0.911 | 3.06 | 86 | 77 |  | 0.73^*^ | 0.606 to 0.833 | 1.48 | 44 | 98 |  | -0.15 | 0.19 | -0.15 |
| /height | (m) | 0.73^*^ | 0.531 to 0.875 | 33 | 86 | 77 |  | 0.69^*^ | 0.584 to 0.779 | 33 | 88 | 48 |  | 0.12 | 0.21^‡^ | 0.21^‡^ |
| /height^3.05^ (m) |  | 0.68 | 0.484 to 0.841 | 12.2 | 86 | 64 |  | 0.68^*^ | 0.557 to 0.794 | 8.44 | 60 | 83 |  | 0.02 | -0.03 | -0.03 |
| /forearm circumference | (cm) | 0.75^*^ | 0.552 to 0.889 | 2.16 | 86 | 77 |  | 0.70^*^ | 0.577 to 0.810 | 1.38 | 60 | 75 |  | 0.01 | 0.27^‡^ | -0.16 |
| /forearm circumference^0.38^ |  | 0.75^*^ | 0.552 to 0.889 | 16.1 | 86 | 77 |  | 0.69^*^ | 0.560 to 0.796 | 11.2 | 76 | 58 |  | 0.11 | 0.3^‡^ | -0.02 |
| /calf circumference |  | 0.74^*^ | 0.545 to 0.884 | 1.65 | 86 | 77 |  | 0.70^*^ | 0.573 to 0.807 | 1.06 | 72 | 68 |  | 0.02 | 0.26^‡^ | -0.02 |
| /calf circumference^1.10^ |  | 0.75^*^ | 0.552 to 0.889 | 1.14 | 86 | 77 |  | 0.71^*^ | 0.580 to 0.813 | 0.7 | 68 | 73 |  | 0.01 | 0.26^‡^ | -0.04 |
| /chest circumference |  | 0.76^*^ | 0.566 to 0.898 | 0.64 | 86 | 73 |  | 0.71^*^ | 0.580 to 0.813 | 0.4 | 72 | 65 |  | -0.03 | 0.26^‡^ | -0.15 |
| /chest circumference^0.56^ |  | 0.75^*^ | 0.552 to 0.889 | 4.62 | 86 | 77 |  | 0.69^*^ | 0.566 to 0.801 | 3.06 | 76 | 60 |  | 0.06 | 0.29^‡^ | -0.05 |
| /waist circumference |  | 0.78^*^ | 0.587 to 0.911 | 0.73 | 100 | 59 |  | 0.72^*^ | 0.591 to 0.821 | 0.37 | 60 | 80 |  | -0.11 | 0.26^‡^ | -0.24^‡^ |
| /waist circumference^0.28^ |  | 0.75^*^ | 0.552 to 0.889 | 16.5 | 86 | 77 |  | 0.69^*^ | 0.563 to 0.799 | 11.4 | 76 | 58 |  | 0.09 | 0.31^‡^ | 0.01 |
| /knee height |  | 0.73^*^ | 0.538 to 0.880 | 1.02 | 86 | 77 |  | 0.68^*^ | 0.554 to 0.792 | 0.78 | 76 | 60 |  | 0.10 | 0.22^‡^ | 0.15 |
| /knee height^2.28^ |  | 0.71 | 0.510 to 0.861 | 0.0065 | 86 | 77 |  | 0.68^*^ | 0.554 to 0.792 | 0.0041 | 52 | 83 |  | 0.01 | 0.09 | 0.24^‡^ |
| /half arm span |  | 0.72^*^ | 0.524 to 0.870 | 0.65 | 86 | 77 |  | 0.68^*^ | 0.547 to 0.786 | 0.48 | 76 | 60 |  | 0.13 | 0.23^‡^ | 0.26^‡^ |
| /half arm span^2.73^ |  | 0.64 | 0.438 to 0.806 | 0.48 | 76 | 60 |  | 0.67^*^ | 0.545 to 0.784 | 0.00026 | 76 | 58 |  | 0.05 | 0.05 | 0.14 |
| /triceps skinfold | (mm) | 0.81^†^ | 0.624 to 0.932 | 4.22 | 86 | 68 |  | 0.70^*^ | 0.575 to 0.809 | 1.4 | 60 | 75 |  | -0.24^‡^ | 0.26^‡^ | 0.07 |
| /triceps skinfold^0.18^ |  | 0.77^*^ | 0.580 to 0.907 | 36.5 | 86 | 77 |  | 0.69^*^ | 0.564 to 0.799 | 21.6 | 76 | 60 |  | 0.09 | 0.34^‡^ | 0.10 |
| /biceps skinfold |  | 0.71 | 0.507 to 0.858 | 7.24 | 71 | 68 |  | 0.76^†^ | 0.637 to 0.856 | 2.5 | 80 | 65 |  | -0.25^‡^ | 0.24^‡^ | -0.38^§^ |
| /biceps skinfold^0.08^ |  | 0.77^*^ | 0.573 to 0.902 | 48.9 | 86 | 77 |  | 0.69^*^ | 0.566 to 0.801 | 31.3 | 76 | 60 |  | 0.12 | 0.32^‡^ | -0.17 |
| /thigh skinfold |  | 0.81^*^ | 0.624 to 0.932 | 2.95 | 71 | 86 |  | 0.79^†^ | 0.668 to 0.879 | 1.18 | 76 | 73 |  | -0.09 | 0.31^‡^ | -0.26^‡^ |
| /thigh skinfold^0.03^ |  | 0.75^*^ | 0.559 to 0.893 | 51.1 | 86 | 77 |  | 0.68^*^ | 0.553 to 0.790 | 34.1 | 72 | 63 |  | 0.16 | 0.33^‡^ | -0.04 |
| /medial calf skinfold |  | 0.83^†^ | 0.646 to 0.944 | 4.61 | 86 | 73 |  | 0.77^†^ | 0.646 to 0.863 | 1.6 | 76 | 70 |  | -0.09 | 0.23^‡^ | -0.38^§^ |
| /medial calf skinfold^0.02^ |  | 0.75^*^ | 0.559 to 0.893 | 53.6 | 86 | 77 |  | 0.68^*^ | 0.551 to 0.789 | 37.1 | 76 | 60 |  | 0.17 | 0.32^‡^ | -0.02 |
| /biacromial breadth | (mm) | 0.73^*^ | 0.531 to 0.875 | 1.42 | 86 | 77 |  | 0.68^*^ | 0.552 to 0.789 | 1.07 | 76 | 58 |  | 0.08 | 0.27^‡^ | 0.13 |
| /biacromial breadth^1.67^ |  | 0.71 | 0.510 to 0.861 | 0.12 | 86 | 77 |  | 0.68^*^ | 0.550 to 0.788 | 0.11 | 84 | 50 |  | 0.02 | 0.22^‡^ | 0.26^‡^ |
| /bitrochanteric breadth |  | 0.73^*^ | 0.538 to 0.880 | 1.72 | 86 | 77 |  | 0.70^*^ | 0.572 to 0.806 | 1.16 | 76 | 60 |  | 0.08 | 0.27^‡^ | 0.01 |
| /bitrochanteric breadth^0.69^ |  | 0.74^*^ | 0.545 to 0.884 | 5.1 | 86 | 77 |  | 0.69^*^ | 0.567 to 0.802 | 3.48 | 76 | 60 |  | 0.11 | 0.28^‡^ | -0.04 |
| /bimalleolar breadth |  | 0.73^*^ | 0.531 to 0.875 | 8.77 | 86 | 77 |  | 0.70^*^ | 0.575 to 0.808 | 5.77 | 76 | 65 |  | 0.10 | 0.24^‡^ | 0.12 |
| /bimalleolar breadth^1.20^ |  | 0.71^*^ | 0.517 to 0.865 | 6.01 | 86 | 68 |  | 0.70^*^ | 0.577 to 0.810 | 3.93 | 76 | 65 |  | 0.08 | 0.22^‡^ | 0.13 |
| /elbow breadth |  | 0.73^*^ | 0.538 to 0.880 | 9.36 | 86 | 73 |  | 0.70^*^ | 0.569 to 0.804 | 6.57 | 76 | 63 |  | 0.05 | 0.28^‡^ | -0.09 |
| /elbow breadth^0.10^ |  | 0.75^*^ | 0.559 to 0.893 | 47.2 | 86 | 77 |  | 0.68^*^ | 0.548 to 0.786 | 32.7 | 76 | 60 |  | 0.16 | 0.32^‡^ | -0.10 |
| /wrist breadth |  | 0.79^*^ | 0.602 to 0.919 | 11.5 | 100 | 64 |  | 0.66^*^ | 0.536 to 0.776 | 7.33 | 72 | 58 |  | 0.07 | 0.29^‡^ | -0.05 |
| /wrist breadth^0.44^ |  | 0.75^*^ | 0.559 to 0.893 | 26.1 | 86 | 77 |  | 0.67^*^ | 0.542 to 0.782 | 18.4 | 72 | 63 |  | 0.13 | 0.31^‡^ | 0.03 |
| /chest breadth |  | 0.73^*^ | 0.538 to 0.880 | 1.85 | 86 | 77 |  | 0.66^*^ | 0.532 to 0.773 | 1.44 | 76 | 55 |  | 0.05 | 0.27^‡^ | 0.02 |
| /chest breadth^0.68^ |  | 0.73^*^ | 0.531 to 0.875 | 5.45 | 86 | 77 |  | 0.66^*^ | 0.536 to 0.776 | 4.06 | 76 | 58 |  | 0.09 | 0.29^‡^ | 0.06 |
| /body mass*height | (kg*m) | 0.73^*^ | 0.538 to 0.880 | 0.52 | 86 | 64 |  | 0.75^†^ | 0.627 to 0.849 | 0.33 | 68 | 78 |  | -0.33^‡^ | 0.02 | 0.10 |
| /(body mass*height)^0.48^ |  | 0.75^*^ | 0.552 to 0.889 | 5.83 | 86 | 77 |  | 0.72^*^ | 0.589 to 0.820 | 4.06 | 76 | 65 |  | -0.07 | 0.18 | 0.14 |
| /SA | (m²) | 0.75^*^ | 0.552 to 0.889 | 31.6 | 86 | 77 |  | 0.72^*^ | 0.593 to 0.823 | 21.2 | 72 | 68 |  | -0.09 | 0.18 | -0.29^‡^ |
| /SA^0.93^ |  | 0.75^*^ | 0.559 to 0.893 | 33 | 86 | 77 |  | 0.72^*^ | 0.589 to 0.820 | 22.7 | 76 | 65 |  | -0.07 | 0.19 | -0.03 |
| /MAMC | (cm) | 0.77^*^ | 0.573 to 0.902 | 2.42 | 86 | 73 |  | 0.72^*^ | 0.592 to 0.822 | 1.54 | 64 | 75 |  | -0.03 | 0.26^‡^ | -0.11 |
| /MAMC^0.18^ |  | 0.75^*^ | 0.559 to 0.893 | 32 | 86 | 77 |  | 0.68^*^ | 0.557 to 0.794 | 22.5 | 76 | 60 |  | 0.14 | 0.31^‡^ | -0.09 |
| /CAMA | (cm²) | 0.71^*^ | 0.517 to 0.865 | 2.03 | 100 | 41 |  | 0.73^*^ | 0.602 to 0.830 | 0.9 | 52 | 93 |  | -0.26^‡^ | 0.14 | -0.24^‡^ |
| /CAMA^0.08^ |  | 0.75^*^ | 0.559 to 0.893 | 41.7 | 86 | 77 |  | 0.69^*^ | 0.558 to 0.795 | 28.5 | 72 | 63 |  | 0.13 | 0.31^‡^ | 0.04 |
| /FFM_(LEAN et al., 1996)_ | (kg) | 0.76^*^ | 0.566 to 0.898 | 1.11 | 86 | 77 |  | 0.72^*^ | 0.592 to 0.822 | 1 | 72 | 68 |  | -0.09 | 0.18 | -0.18 |
| /FFM^0.88^_(LEAN et al., 1996)_ |  | 0.75^*^ | 0.559 to 0.893 | 1.77 | 86 | 77 |  | 0.71^*^ | 0.583 to 0.815 | 1.53 | 76 | 65 |  | -0.06 | 0.20 | 0.20 |
| /left arm LST | (g) | 0.64 | 0.438 to 0.806 | 0.026 | 86 | 59 |  | 0.70^*^ | 0.568 to 0.803 | 0.021 | 52 | 88 |  | -0.18 | 0.14 | 0.05 |
| /left arm LST^0.26^ |  | 0.72^*^ | 0.524 to 0.870 | 7.77 | 86 | 77 |  | 0.69^*^ | 0.560 to 0.796 | 5.86 | 72 | 63 |  | 0.08 | 0.28^‡^ | 0.07 |
| /right arm LST |  | 0.62 | 0.425 to 0.795 | 0.026 | 100 | 45 |  | 0.70^*^ | 0.578 to 0.811 | 0.013 | 40 | 98 |  | -0.17 | 0.18 | -0.30^‡^ |
| /right arm LST^0.22^ |  | 0.73^*^ | 0.538 to 0.880 | 10.1 | 86 | 77 |  | 0.69^*^ | 0.559 to 0.795 | 7.44 | 72 | 63 |  | 0.09 | 0.3^‡^ | 0.07 |
| /left leg LST |  | 0.68 | 0.477 to 0.836 | 0.0079 | 86 | 64 |  | 0.68^*^ | 0.553 to 0.790 | 0.0048 | 44 | 93 |  | -0.18 | 0.08 | -0.15 |
| /left leg LST^0.64^ |  | 0.72^*^ | 0.524 to 0.870 | 0.19 | 86 | 77 |  | 0.69^*^ | 0.560 to 0.796 | 0.12 | 44 | 88 |  | -0.05 | 0.17 | 0.19 |
| /right leg LST |  | 0.65 | 0.451 to 0.816 | 0.0079 | 86 | 55 |  | 0.69^*^ | 0.561 to 0.797 | 0.005 | 44 | 93 |  | -0.15 | 0.13 | -0.14 |
| /right leg LST^0.58^ |  | 0.73^*^ | 0.531 to 0.875 | 0.33 | 86 | 77 |  | 0.69^*^ | 0.562 to 0.798 | 0.21 | 48 | 83 |  | -0.01 | 0.21^‡^ | 0.01 |
| /arms LST |  | 0.63 | 0.432 to 0.801 | 0.012 | 86 | 55 |  | 0.70^*^ | 0.572 to 0.806 | 0.009 | 52 | 85 |  | -0.18 | 0.17 | -0.18 |
| /arms LST^0.26^ |  | 0.72^*^ | 0.524 to 0.870 | 6.38 | 86 | 77 |  | 0.69^*^ | 0.561 to 0.797 | 4.76 | 72 | 63 |  | 0.08 | 0.29^‡^ | 0.01 |
| /legs LST |  | 0.68 | 0.477 to 0.836 | 0.0039 | 86 | 59 |  | 0.68^*^ | 0.556 to 0.793 | 0.0024 | 44 | 93 |  | -0.16 | 0.10 | -0.17 |
| /legs LST^0.64^ |  | 0.73^*^ | 0.531 to 0.875 | 0.12 | 86 | 77 |  | 0.69^*^ | 0.559 to 0.795 | 0.08 | 44 | 88 |  | -0.04 | 0.18 | 0.18 |
| /ASM | (kg) | 0.66 | 0.457 to 0.821 | 2.99 | 86 | 55 |  | 0.69^*^ | 0.561 to 0.797 | 1.74 | 44 | 93 |  | -0.17 | 0.12 | -0.17 |
| /ASM^0.56^ |  | 0.72^*^ | 0.524 to 0.870 | 10.7 | 86 | 77 |  | 0.69^*^ | 0.565 to 0.801 | 5.68 | 36 | 98 |  | -0.02 | 0.21^‡^ | -0.01 |
| /ASM/height² | (kg/m²) | 0.73^*^ | 0.538 to 0.880 | 8.47 | 86 | 73 |  | 0.70^*^ | 0.568 to 0.803 | 4.88 | 48 | 88 |  | -0.06 | 0.34^‡^ | -0.28^‡^ |
| /ASM/height²^(0.23)^ |  | 0.73^*^ | 0.538 to 0.880 | 36.3 | 86 | 77 |  | 0.68^*^ | 0.554 to 0.791 | 22.4 | 60 | 73 |  | 0.12 | 0.33^‡^ | -0.10 |
| /FFM_(BAUMGARTNER et al., 1991)_ | (kg) | 0.78^*^ | 0.584 to 0.914 | 1.13 | 83 | 73 |  | 0.75^†^ | 0.620 to 0.845 | 0.83 | 76 | 64 |  | -0.14 | 0.20 | -0.03 |
| /FFM^0.67^_(BAUMGARTNER et al., 1991)_ |  | 0.76^*^ | 0.559 to 0.898 | 3.94 | 83 | 77 |  | 0.72^*^ | 0.592 to 0.823 | 2.91 | 76 | 64 |  | -0.03 | 0.24^‡^ | 0.16 |
| /FFM_(DXA)_ |  | 0.70 | 0.504 to 0.856 | 1.16 | 86 | 73 |  | 0.70^*^ | 0.575 to 0.808 | 0.67 | 44 | 93 |  | -0.17 | 0.11 | -0.08 |
| /FFM^0.38^_(DXA)_ |  | 0.72^*^ | 0.524 to 0.870 | 12.7 | 86 | 77 |  | 0.69^*^ | 0.562 to 0.798 | 9.48 | 72 | 60 |  | 0.04 | 0.24^‡^ | 0.02 |
|  |  |  |  |  |  |  |  |  |  |  |  |  |  |  |  |  |
| ***PT (Nm)*** | |  |  |  |  |  |  |  |  |  |  |  |  |  |  |  |
| Non-normalized |  | 0.93^†^ | 0.769 to 0.991 | 85.4 | 86 | 95 |  | 0.74^†^ | 0.619 to 0.843 | 66.6 | 64 | 75 |  | 0.20 | 0.42^§^ | - |
| /body mass | (kg) | 0.90^†^ | 0.734 to 0.981 | 1.26 | 86 | 91 |  | 0.84^†^ | 0.726 to 0.918 | 0.93 | 72 | 88 |  | -0.32^‡^ | 0.17 | -0.32^‡^ |
| /body mass^0.37^ |  | 0.93^†^ | 0.769 to 0.991 | 18.2 | 86 | 95 |  | 0.80^†^ | 0.677 to 0.885 | 16.2 | 80 | 68 |  | 0.01 | 0.33^‡^ | 0.01 |
| /body mass^0.67 (DAVIES; DALSKY, 1997)^ |  | 0.93^†^ | 0.769 to 0.991 | 5.06 | 86 | 95 |  | 0.82^†^ | 0.704 to 0.904 | 3.71 | 68 | 88 |  | -0.15 | 0.26^‡^ | -0.15 |
| /body mass^0.72 (DAVIES; DALSKY, 1997)^ |  | 0.94^†^ | 0.778 to 0.993 | 4.1 | 86 | 95 |  | 0.82^†^ | 0.710 to 0.907 | 3.14 | 72 | 85 |  | -0.18 | 0.24^‡^ | -0.18 |
| /body mass^0.74 (DAVIES; DALSKY, 1997)^ |  | 0.93^†^ | 0.769 to 0.991 | 3.77 | 86 | 95 |  | 0.82^†^ | 0.710 to 0.907 | 2.87 | 72 | 85 |  | -0.19 | 0.24^‡^ | -0.19 |
| /body mass^0.67 (JARIC, 2003)^ |  | 0.93^†^ | 0.769 to 0.991 | 5.06 | 86 | 95 |  | 0.82^†^ | 0.704 to 0.904 | 3.71 | 68 | 88 |  | -0.15 | 0.26^‡^ | -0.15 |
| /height | (m) | 0.93^†^ | 0.769 to 0.991 | 54.1 | 86 | 95 |  | 0.74^†^ | 0.620 to 0.843 | 44.1 | 64 | 75 |  | 0.14 | 0.30^‡^ | 0.30^‡^ |
| /height^3.27^ |  | 0.86^†^ | 0.677 to 0.958 | 19.2 | 100 | 77 |  | 0.74^†^ | 0.617 to 0.842 | 17.4 | 72 | 65 |  | 0.01 | 0.01 | 0.01 |
| /forearm circumference | (cm) | 0.94^†^ | 0.787 to 0.995 | 3.88 | 100 | 82 |  | 0.80^†^ | 0.683 to 0.890 | 2.59 | 60 | 88 |  | 0.01 | 0.36^§^ | -0.09 |
| /forearm circumference^0.59^ |  | 0.95^†^ | 0.796 to 0.996 | 14.9 | 100 | 86 |  | 0.78^†^ | 0.664 to 0.876 | 12.8 | 92 | 55 |  | 0.08 | 0.38^§^ | 0.02 |
| /calf circumference |  | 0.90^†^ | 0.734 to 0.981 | 2.46 | 86 | 91 |  | 0.79^†^ | 0.676 to 0.884 | 2.13 | 84 | 65 |  | 0.02 | 0.34^‡^ | -0.01 |
| /calf circumference^1.18^ |  | 0.90^†^ | 0.734 to 0.981 | 1.31 | 86 | 91 |  | 0.80^†^ | 0.686 to 0.891 | 1.2 | 88 | 63 |  | -0.01 | 0.32^‡^ | -0.05 |
| /chest circumference |  | 0.90^†^ | 0.726 to 0.978 | 0.9 | 86 | 91 |  | 0.79^†^ | 0.676 to 0.884 | 0.67 | 64 | 83 |  | -0.03 | 0.34^‡^ | -0.12 |
| /chest circumference^0.72^ |  | 0.90^†^ | 0.734 to 0.981 | 3.16 | 86 | 91 |  | 0.78^†^ | 0.663 to 0.876 | 2.27 | 60 | 85 |  | 0.03 | 0.36^§^ | -0.04 |
| /waist circumference |  | 0.90^†^ | 0.726 to 0.978 | 0.93 | 86 | 91 |  | 0.79^†^ | 0.670 to 0.880 | 0.71 | 68 | 83 |  | -0.13 | 0.33^‡^ | -0.23^‡^ |
| /waist circumference^0.40^ |  | 0.93^†^ | 0.769 to 0.991 | 13.8 | 86 | 95 |  | 0.77^†^ | 0.648 to 0.865 | 11.4 | 72 | 73 |  | 0.07 | 0.38^§^ | -0.01 |
| /knee height |  | 0.94^†^ | 0.778 to 0.993 | 1.83 | 100 | 86 |  | 0.75^†^ | 0.624 to 0.847 | 1.44 | 68 | 73 |  | 0.12 | 0.30^‡^ | 0.14 |
| /knee height^1.82^ |  | 0.90^†^ | 0.734 to 0.981 | 0.068 | 100 | 86 |  | 0.74^†^ | 0.617 to 0.842 | 0.053 | 64 | 75 |  | 0.05 | 0.21^‡^ | 0.02 |
| /half arm span |  | 0.91^†^ | 0.743 to 0.984 | 1.04 | 86 | 91 |  | 0.75^†^ | 0.627 to 0.849 | 0.9 | 68 | 70 |  | 0.15 | 0.31^‡^ | 0.11 |
| /half arm span^1.62^ |  | 0.88^†^ | 0.709 to 0.972 | 0.076 | 100 | 73 |  | 0.75^†^ | 0.631 to 0.852 | 0.066 | 84 | 58 |  | 0.11 | 0.24^‡^ | 0.01 |
| /triceps skinfold | (mm) | 0.86^†^ | 0.677 to 0.958 | 5.26 | 86 | 86 |  | 0.78^†^ | 0.660 to 0.873 | 2.45 | 64 | 85 |  | -0.25^‡^ | 0.26^‡^ | -0.57^§^ |
| /triceps skinfold^0.15^ |  | 0.93^†^ | 0.769 to 0.991 | 55.6 | 86 | 95 |  | 0.76^†^ | 0.641 to 0.860 | 47.6 | 80 | 63 |  | 0.12 | 0.42^§^ | -0.12 |
| /biceps skinfold |  | 0.79^*^ | 0.602 to 0.919 | 16.8 | 100 | 59 |  | 0.82^†^ | 0.704 to 0.904 | 5.3 | 92 | 63 |  | -0.27^‡^ | 0.25^‡^ | -0.56^§^ |
| /biceps skinfold^0.06^ |  | 0.93^†^ | 0.769 to 0.991 | 76.1 | 86 | 95 |  | 0.75^†^ | 0.631 to 0.852 | 71.9 | 88 | 53 |  | 0.16 | 0.41^§^ | -0.14 |
| /thigh skinfold |  | 0.92^†^ | 0.751 to 0.986 | 5.31 | 100 | 86 |  | 0.82^†^ | 0.708 to 0.907 | 1.85 | 72 | 83 |  | -0.11 | 0.32^‡^ | -0.57^§^ |
| /thigh skinfold^-0.10^ |  | 0.90^†^ | 0.734 to 0.981 | 115 | 86 | 91 |  | 0.73^†^ | 0.601 to 0.829 | 105 | 76 | 60 |  | 0.23^‡^ | 0.39^§^ | -0.06 |
| /medial calf skinfold |  | 0.92^†^ | 0.760 to 0.988 | 7.21 | 86 | 95 |  | 0.83^†^ | 0.720 to 0.914 | 2.5 | 72 | 85 |  | -0.10 | 0.25^‡^ | -0.51^§^ |
| /medial calf skinfold^0.003^ |  | 0.93^†^ | 0.769 to 0.991 | 84.8 | 86 | 95 |  | 0.74^†^ | 0.620 to 0.843 | 66 | 64 | 75 |  | 0.20 | 0.41^§^ | -0.04 |
| /biacromial breadth | (mm) | 0.90^†^ | 0.726 to 0.978 | 2.56 | 100 | 73 |  | 0.76^†^ | 0.642 to 0.861 | 2.04 | 84 | 60 |  | 0.1 | 0.35^§^ | 0.15 |
| /biacromial breadth^2.15^ |  | 0.82^†^ | 0.631 to 0.936 | 0.038 | 86 | 68 |  | 0.77^†^ | 0.647 to 0.864 | 0.03 | 76 | 73 |  | -0.01 | 0.27^‡^ | -0.04 |
| /bitrochanteric breadth |  | 0.93^†^ | 0.769 to 0.991 | 2.56 | 86 | 95 |  | 0.79^†^ | 0.666 to 0.877 | 2.13 | 76 | 73 |  | 0.08 | 0.35^§^ | -0.08 |
| /bitrochanteric breadth^0.50^ |  | 0.93^†^ | 0.769 to 0.991 | 14.8 | 86 | 95 |  | 0.77^†^ | 0.647 to 0.864 | 14.1 | 88 | 58 |  | 0.14 | 0.38^§^ | 0.01 |
| /bimalleolar breadth |  | 0.88^†^ | 0.701 to 0.969 | 13.6 | 86 | 86 |  | 0.79^†^ | 0.666 to 0.877 | 12.0 | 84 | 65 |  | 0.10 | 0.31^‡^ | 0.03 |
| /bimalleolar breadth^1.54^ |  | 0.81^*^ | 0.616 to 0.928 | 5.04 | 86 | 73 |  | 0.80^†^ | 0.681 to 0.888 | 4.64 | 92 | 60 |  | 0.06 | 0.26^‡^ | -0.07 |
| /elbow breadth |  | 0.90^†^ | 0.726 to 0.978 | 14 | 86 | 86 |  | 0.79^†^ | 0.674 to 0.884 | 13.9 | 88 | 63 |  | 0.07 | 0.37^§^ | -0.20 |
| /elbow breadth^0.10^ |  | 0.93^†^ | 0.769 to 0.991 | 70.6 | 86 | 95 |  | 0.75^†^ | 0.624 to 0.847 | 56.6 | 64 | 75 |  | 0.18 | 0.41^§^ | 0.01 |
| /wrist breadth |  | 0.93^†^ | 0.769 to 0.991 | 15.9 | 86 | 95 |  | 0.76^†^ | 0.636 to 0.856 | 14.2 | 68 | 73 |  | 0.09 | 0.38^§^ | -0.07 |
| /wrist breadth^0.80^ |  | 0.93^†^ | 0.769 to 0.991 | 22.1 | 86 | 95 |  | 0.76^†^ | 0.635 to 0.855 | 19.7 | 72 | 73 |  | 0.11 | 0.38^§^ | -0.03 |
| /chest breadth |  | 0.89^†^ | 0.718 to 0.975 | 3.33 | 100 | 73 |  | 0.75^†^ | 0.625 to 0.848 | 2.2 | 56 | 83 |  | 0.07 | 0.37^§^ | 0.05 |
| /chest breadth^1.40^ |  | 0.88^†^ | 0.701 to 0.969 | 0.85 | 100 | 73 |  | 0.74^†^ | 0.611 to 0.837 | 0.77 | 84 | 55 |  | 0.02 | 0.34^‡^ | -0.04 |
| /body mass*height | (kg*m) | 0.84^†^ | 0.654 to 0.947 | 0.8 | 86 | 77 |  | 0.84^†^ | 0.729 to 0.920 | 0.6 | 72 | 88 |  | -0.38^§^ | 0.04 | -0.32^‡^ |
| /(body mass*height)^0.43^ |  | 0.94^†^ | 0.787 to 0.995 | 13 | 100 | 82 |  | 0.80^†^ | 0.681 to 0.888 | 10.5 | 84 | 65 |  | -0.06 | 0.26^‡^ | 0.01 |
| /(body mass*height)^0.97 (SEGAL et al., 2008)^ |  | 0.86^†^ | 0.677 to 0.958 | 0.9 | 86 | 77 |  | 0.84^†^ | 0.729 to 0.920 | 0.68 | 72 | 88 |  | -0.37^§^ | 0.05 | -0.31^‡^ |
| /SA | (m²) | 0.94^†^ | 0.787 to 0.995 | 56.9 | 100 | 82 |  | 0.81^†^ | 0.692 to 0.896 | 36.3 | 64 | 88 |  | -0.10 | 0.24^‡^ | -0.04 |
| /SA^0.83^ |  | 0.94^†^ | 0.778 to 0.993 | 53.8 | 86 | 95 |  | 0.80^†^ | 0.680 to 0.887 | 50 | 84 | 65 |  | -0.05 | 0.28^‡^ | 0.01 |
| /MAMC | (cm) | 0.95^†^ | 0.796 to 0.996 | 4.11 | 100 | 86 |  | 0.80^†^ | 0.677 to 0.885 | 2.82 | 68 | 88 |  | -0.04 | 0.34^‡^ | -0.21^‡^ |
| /MAMC^0.25^ |  | 0.94^†^ | 0.778 to 0.993 | 45.9 | 100 | 82 |  | 0.77^†^ | 0.643 to 0.861 | 32.9 | 72 | 70 |  | 0.14 | 0.40^§^ | 0.08 |
| /CAMA | (cm²) | 0.96^†^ | 0.806 to 0.998 | 2.66 | 100 | 82 |  | 0.80^†^ | 0.685 to 0.891 | 2.49 | 84 | 73 |  | -0.31^‡^ | 0.18 | -0.58^§^ |
| /CAMA^0.11^ |  | 0.94^†^ | 0.778 to 0.993 | 68.5 | 100 | 82 |  | 0.77^†^ | 0.643 to 0.861 | 48.7 | 72 | 70 |  | 0.14 | 0.40^§^ | 0.10 |
| /FFM_(LEAN et al., 1996)_ | (kg) | 0.93^†^ | 0.769 to 0.991 | 1.79 | 86 | 95 |  | 0.81^†^ | 0.697 to 0.899 | 1.84 | 76 | 80 |  | -0.11 | 0.23^‡^ | -0.10 |
| /FFM^0.53^_(LEAN et al., 1996)_ |  | 0.94^†^ | 0.778 to 0.993 | 10.7 | 86 | 95 |  | 0.79^†^ | 0.674 to 0.884 | 11.2 | 84 | 65 |  | 0.04 | 0.32^‡^ | 0.06 |
| /left arm LST | (g) | 0.81^*^ | 0.616 to 0.928 | 0.047 | 86 | 68 |  | 0.76^†^ | 0.641 to 0.860 | 0.047 | 76 | 75 |  | -0.20 | 0.19 | -0.35^§^ |
| /left arm LST^0.32^ |  | 0.91^†^ | 0.743 to 0.984 | 7.65 | 86 | 91 |  | 0.77^†^ | 0.646 to 0.863 | 6.72 | 72 | 70 |  | 0.07 | 0.36^§^ | 0.09 |
| /right arm LST |  | 0.80^*^ | 0.609 to 0.924 | 0.034 | 71 | 86 |  | 0.78^†^ | 0.662 to 0.875 | 0.036 | 68 | 83 |  | -0.18 | 0.21^‡^ | -0.44^§^ |
| /right arm LST^0.09^ |  | 0.93^†^ | 0.769 to 0.991 | 41.8 | 86 | 95 |  | 0.76^†^ | 0.635 to 0.855 | 38.8 | 80 | 63 |  | 0.16 | 0.40^§^ | 0.10 |
| /left leg LST |  | 0.82^†^ | 0.631 to 0.936 | 0.015 | 100 | 55 |  | 0.76^†^ | 0.636 to 0.856 | 0.012 | 68 | 80 |  | -0.19 | 0.13 | -0.25^‡^ |
| /left leg LST^0.43^ |  | 0.92^†^ | 0.760 to 0.988 | 2.26 | 100 | 77 |  | 0.77^†^ | 0.648 to 0.865 | 1.83 | 76 | 70 |  | 0.03 | 0.30^‡^ | 0.05 |
| /right leg LST |  | 0.77^*^ | 0.580 to 0.907 | 0.015 | 100 | 50 |  | 0.78^†^ | 0.662 to 0.875 | 0.013 | 76 | 78 |  | -0.16 | 0.18 | -0.21 |
| /right leg LST^0.48^ |  | 0.90^†^ | 0.734 to 0.981 | 1.39 | 100 | 73 |  | 0.78^†^ | 0.656 to 0.870 | 1.16 | 80 | 70 |  | 0.02 | 0.31^‡^ | 0.05 |
| /arms LST |  | 0.81^*^ | 0.616 to 0.928 | 0.018 | 71 | 82 |  | 0.78^†^ | 0.656 to 0.870 | 0.022 | 76 | 78 |  | -0.19 | 0.21 | -0.38^§^ |
| /arms LST^0.21^ |  | 0.92^†^ | 0.751 to 0.986 | 14.8 | 86 | 95 |  | 0.77^†^ | 0.651 to 0.867 | 14.0 | 80 | 63 |  | 0.11 | 0.38^§^ | 0.10 |
| /legs LST |  | 0.81^*^ | 0.616 to 0.928 | 0.0063 | 71 | 82 |  | 0.77^†^ | 0.651 to 0.867 | 0.0065 | 76 | 78 |  | -0.18 | 0.15 | -0.21^‡^ |
| /legs LST^0.47^ |  | 0.92^†^ | 0.751 to 0.986 | 1.07 | 100 | 73 |  | 0.77^†^ | 0.652 to 0.868 | 0.87 | 76 | 70 |  | 0.02 | 0.30^‡^ | 0.05 |
| /ASM | (kg) | 0.81^*^ | 0.624 to 0.932 | 4.66 | 71 | 86 |  | 0.77^†^ | 0.652 to 0.868 | 5.01 | 76 | 78 |  | -0.18 | 0.17 | -0.23^‡^ |
| /ASM^0.42^ |  | 0.91^†^ | 0.743 to 0.984 | 27.2 | 86 | 86 |  | 0.77^†^ | 0.646 to 0.863 | 23.9 | 76 | 70 |  | 0.03 | 0.32^‡^ | 0.07 |
| /ASM/height² | (kg/m²) | 0.88^†^ | 0.701 to 0.969 | 11.9 | 71 | 95 |  | 0.78^†^ | 0.656 to 0.870 | 11.5 | 72 | 78 |  | -0.06 | 0.39^§^ | -0.35^§^ |
| /ASM/height²^(0.02)^ |  | 0.93^†^ | 0.769 to 0.991 | 82.3 | 86 | 95 |  | 0.74^†^ | 0.620 to 0.843 | 64.6 | 64 | 75 |  | 0.19 | 0.41^§^ | 0.06 |
| /FFM_(BAUMGARTNER et al., 1991)_ | (kg) | 0.93^†^ | 0.769 to 0.992 | 1.63 | 83 | 95 |  | 0.85^†^ | 0.734 to 0.924 | 1.6 | 88 | 77 |  | -0.16 | 0.25^‡^ | -0.15 |
| /FFM^0.61^_(BAUMGARTNER et al., 1991)_ |  | 0.92^†^ | 0.759 to 0.990 | 7.54 | 83 | 95 |  | 0.81^†^ | 0.694 to 0.898 | 7.2 | 88 | 67 |  | -0.02 | 0.32^‡^ | 0.01 |
| /FFM_(DXA)_ |  | 0.84^†^ | 0.654 to 0.947 | 2.08 | 100 | 59 |  | 0.79^†^ | 0.672 to 0.882 | 1.89 | 76 | 75 |  | -0.19 | 0.17 | -0.23^‡^ |
| /FFM^0.32^_(DXA)_ |  | 0.93^†^ | 0.769 to 0.991 | 26.1 | 86 | 95 |  | 0.77^†^ | 0.652 to 0.868 | 25.9 | 88 | 55 |  | 0.07 | 0.34^‡^ | 0.09 |

^*^p<0.05 and ^†^p<0.001 (statistically significant AUC)

^‡^p<0.05 and ^§^p<0.001 (statistically significant correlation)

Dependent variable (primary outcome): functional limitation (6MWT<400 m)

*Note*: AUC=area under the curve; CI=confidence interval; *p*=significance; Sens=sensibility; Spe=specificity; SA=surface area of human body; MAMC=mid-arm muscle circumference; CAMA=corrected arm muscle area; AFA=arm fat area; FFM=Fat-free mass; LST=lean soft tissue; ASM=appendicular skeletal muscle mass; DXA=Dual-energy X-ray absorptiometry; 6MWT=six-minute walk test.
